# Building Large DNA Bundles via Controlled Hierarchical Assembly of DNA Tubes

**DOI:** 10.1021/acsnano.3c01342

**Published:** 2023-05-19

**Authors:** Yunlong Zhang, Donglei Yang, Pengfei Wang, Yonggang Ke

**Affiliations:** † Department of Chemistry, Emory University, Atlanta, Georgia 30322, United States; ‡ Institute of Molecular Medicine, Department of Laboratory Medicine, Shanghai Key Laboratory for Nucleic Acid Chemistry and Nanomedicine, Center for DNA Information Storage, Renji Hospital, School of Medicine, Shanghai Jiao Tong University, Shanghai 200127, China; § Wallace H. Coulter Department of Biomedical Engineering, Georgia Institute of Technology and Emory University, Atlanta, Georgia 30322, United States

**Keywords:** DNA nanotechnology, DNA nanotubes, large DNA bundles, hierarchical assembly, reconfigurable

## Abstract

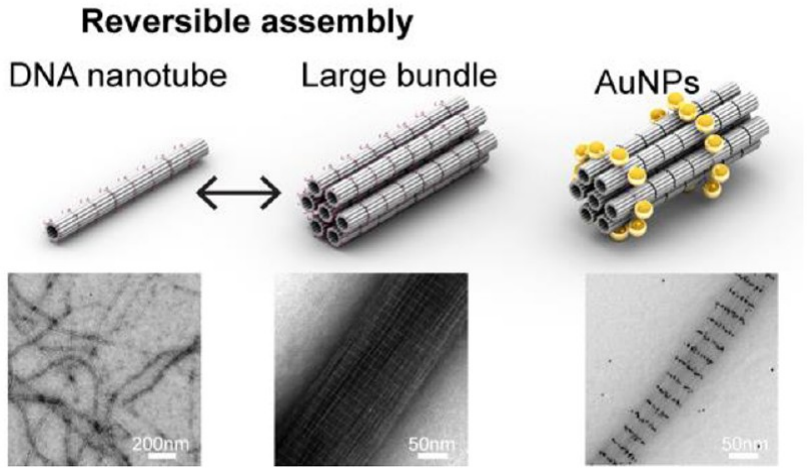

Structural DNA nanotechnology is capable of fabricating designer nanoscale artificial architectures. Developing simple and yet versatile assembly methods to construct large DNA structures of defined spatial features and dynamic capabilities has remained challenging. Herein, we designed a molecular assembly system where DNA tiles can assemble into tubes and then into large one-dimensional DNA bundles following a hierarchical pathway. A cohesive link was incorporated into the tile to induce intertube binding for the formation of DNA bundles. DNA bundles with length of dozens of micrometers and width of hundreds of nanometers were produced, whose assembly was revealed to be collectively determined by cationic strength and linker designs (binding strength, spacer length, linker position, etc.). Furthermore, multicomponent DNA bundles with programmable spatial features and compositions were realized by using various distinct tile designs. Lastly, we implemented dynamic capability into large DNA bundles to realize reversible reconfigurations among tile, tube, and bundles following specific molecular stimulations. We envision this assembly strategy can enrich the toolbox of DNA nanotechnology for rational design of large-size DNA materials of defined features and properties that may be applied to a variety of fields in materials science, synthetic biology, biomedical science, and beyond.

Bundled fiber structures, commonly presented in nature, exhibit numerous critical biological functions including molecule transportation, cell migration, cell division, etc.,^1^ and they also play roles in numerous pathological processes such as tumor progression and metastasis.^2,3^ These natural bundle structures, such as microtubules or muscle fibers, are typically hierarchically assembled from smaller components following prescribed assembling pathways.^4,5^ Extensive efforts have been devoted to building artificial molecular superstructures that can mimic these biological bundle systems from both structural and functional perspectives, which may serve as robust biomimetic models toward a variety of biological applications.^6−8^

Structural DNA nanotechnology represents one of the most robust and versatile molecular assembly methods for the precise fabrication of nanoscale to macroscale designer structures of prescribed properties. DNA nanotechnology utilizes DNA as basic building blocks to self-assemble into artificial structures with programmable physicochemical properties (size, shape, rigidity, surface chemistry, etc.).^9−11^ A variety of large microscale DNA materials including one-dimensional, two-dimensional, and three-dimensional structures have been assembled by using DNA origami^12−18^ or DNA tile^19−26^ as building units. Particularly, DNA double-crossover (DX) tiles have long been used for the construction of long and flexible DNA nanotubes,^27−45^ which bear resemblance to natural occurred fibers and thus are chosen for programmable assembly of large bundles structures in this work. A recent study reported that a high concentration of cations can cause nonspecific bridging of DNA tubes to form DNA bundles.^46^ Nevertheless, compared to DNA-sequence-driven assembly, such a cation-driven process lacks control over bundle formation, including DNA tube’s registration and density, bundle width, bundle surface programmability, and dynamic capability. Furthermore, this cation-bridged DNA bundle assembly requires a high cationic strength (∼40–50 mM Mg^2+^) that can limit its subsequent applications.

Herein, we designed a DNA DX tile flanked with a cohesive linker to induce programmable binding between DNA tubes for the construction of large DNA bundles under moderate cationic strength. Importantly, the cohesive linker provides a chaperoning force to tune assembly parameters toward better control over bundle features including width, compactness, constitution, and reconfigurability. A general design principle of cohesive linkers for bundle assembly was derived from comprehensive studies. We envision that this work can inspire future rational designs on building macroscopic biomimetic materials of diverse functions, and the fabricated DNA bundle structures may find utilities in fields of synthetic biology, materials science, and biomedical science.

## Results and Discussion

### Design, Assembly, and Characterization of DNA Bundles

**Figure 1:**
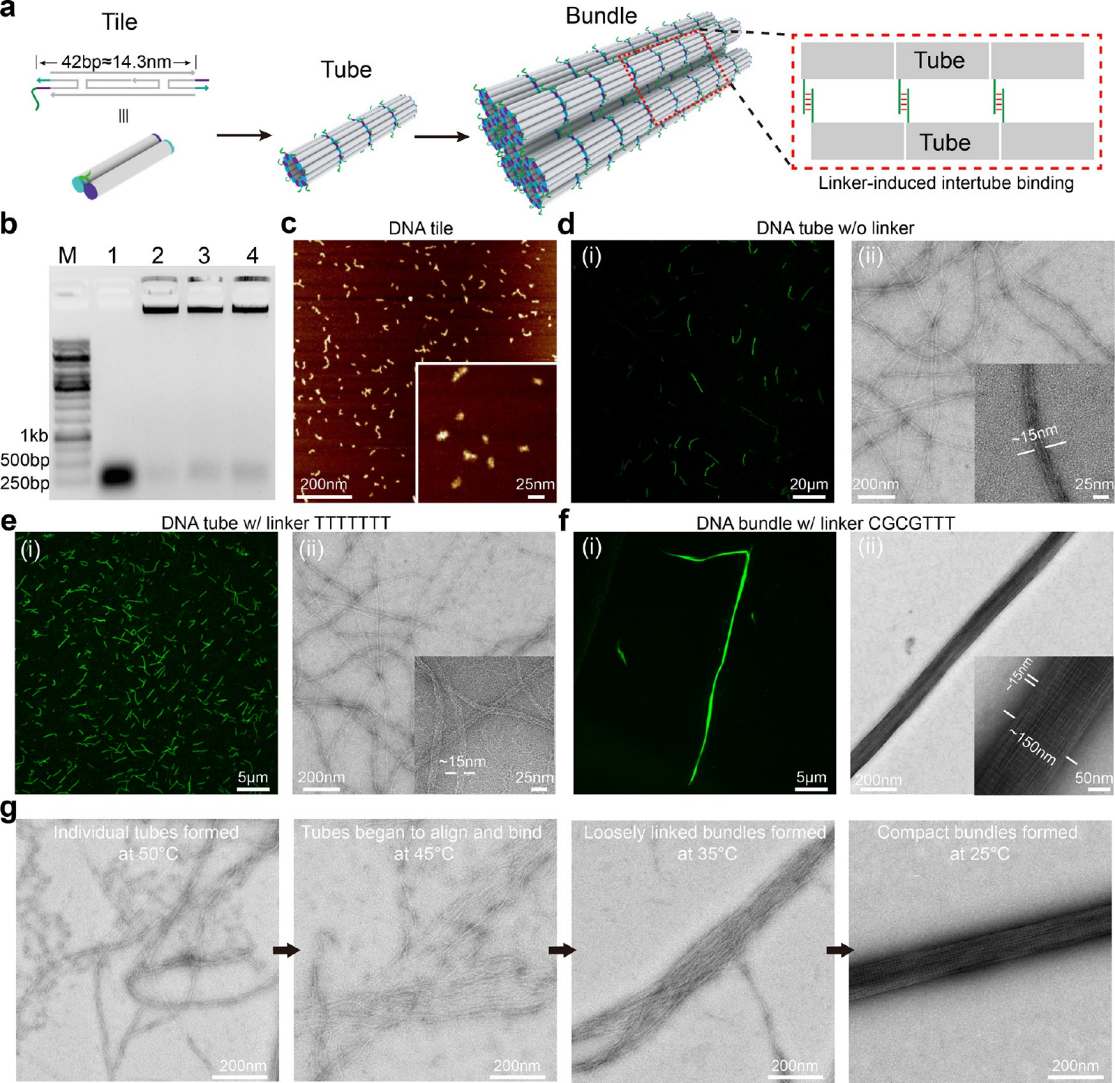
Design, assembly, and characterization of DNA bundles. (a) The hierarchical assembly pathway of DNA bundles from DNA tiles and DNA tubes. DNA double-crossover (DX) tiles first assemble into narrow tubes via sticky-end cohesions (cyan and purple strands), which further assemble into long and wide DNA bundles through DNA linkers (green strands) protruding from the exterior surface of the tubes. (b) Agarose gel electrophoresis analysis of DNA tiles, tubes, and bundles. M: DNA Marker. Lane 1: DNA tiles. Lane 2: DNA tubes assembled from tiles without linkers. Lane 3: DNA tubes assembled from tiles with linker of TTTTTTT (7T). Lane 4: DNA bundles assembled from tiles with linker of CGCGTTT. (c) Atomic force microscopy images of DNA tiles. (d) Confocal laser scanning microscopy (CLSM) (i) and TEM (ii) images of DNA tubes with no linkers. The average width of DNA tubes corresponds to 7–8 tiles along the tube circumference. However, due to the wide distribution of DNA tube length, the total number of DNA tiles in a single DNA tube cannot be accurately measured. (e) CLSM (i) and TEM (ii) images of DNA tubes with linker of 7T. The 7T linker does not allow the complementary binding among linkers, thus causing spatial hindrance and preventing nonspecific DNA nanotube bundling. (f) CLSM (i) and TEM images (ii) of DNA bundles. A cohesive linker of CGCGTTT is designed to induce intertube binding for bundle formation. Three thymidine (T) are added as a spacer to offer a certain degree of flexibility that shall promote the binding between linkers. The periodic white stripes with an interval of ∼15 nm observed on TEM images of DNA bundles (ii, inset) represent the existence of highly oriented DNA linkers after intertube binding. (g) TEM images of self-assembled DNA structures with a linker of CGCGTTT that terminated at designated temperatures during the thermal annealing process for bundle assembly. The hierarchical assembly process was validated as the gradual change from individual tubes to loosely linked bundles, and finally wide, compact, and straight bundles were clearly visualized. Note: Structures were assembled from Tile A unless indicated. For all experiments in this work, 20 mM Mg^2+^ was used for assembly unless otherwise specified.

The overall hierarchical assembly pathway of DNA bundles is illustrated in Figure 1a. The assembly starts from the formation of a DNA double-crossover tile (DX tile) consisting of 5 DNA single strands (Figure S1). The backbone of the DX tile (gray) is 37-bp long, with 5-nucleotide-long sticky ends (cyan and purple) flanking on both sides for assembly into DNA tubes.^30,31,47^ To induce further assembly of DNA tubes, a 7-nucleotide-long cohesive linker is introduced into the tile that protrudes outward from the outer surface of the DNA tubes. Hybridization between DNA linkers enables binding between DNA tubes that induces growth both along and perpendicular to the longitudinal direction of DNA tubes, leading to the formation of long and wide DNA bundles.

The assemblies of DX tiles, DNA tubes, and DNA bundles were annealed in an aqueous buffer containing 20 mM MgCl_2_ and characterized by agarose gel electrophoresis (Figure 1b). DX Tile C is designed with noncomplementary sticky-ends and used to show what individual DX tiles look like (Figure S1). DNA tubes and DNA bundles were assembled from DX tile A (Figure S1) with designated linker sequences. DX tiles showed the expected motility as it migrates fast in the gel. In contrast, DNA tubes and bundles retained in the wells, suggesting the formation of large structures. To unambiguously visualize these structures, atomic force microscopy (AFM), confocal laser scanning microscopy (CLSM), and transmission electron microscopy (TEM) were used for high-resolution imaging. AFM revealed nanoscale rectangular structures whose dimensions agree well with those of individual and loosely connected clusters of DNA tiles (Figure 1c). Micrometer-long DNA tubes with an average width of 14–15 nm (7–8 tiles along the tube circumference) were observed if no linker was incorporated (Figure 1d) or if a TTTTTTT (7T) noncohesive linker was used (Figure 1e), whose width distribution matches well to previous reports of tubes using similar DNA tile designs.^27^ In these cases, binding between DNA tubes was not enabled for bundle assembly. To control bundle assembly in a programmable manner, a cohesive linker of CGCGTTT was rationally designed to induce binding and bundling between DNA tubes to grow into long (∼50 μm) and wide (∼150 nm) bundle structures (Figures 1f, S2, and S3). Our studies revealed that DNA bundles can only be assembled if the cohesive linker is positioned protruding outward (Figure S4). This critical role of the linker position on the DNA tubes determining bundle assembly further validated the proposed assembly mechanism. Individual tubular structures that aligned longitudinally within the bundles can be clearly observed, confirming that these tubes were bundled together (Figure 1f). In addition, periodic white stripes in the transverse direction were observed, which correspond to the DNA linkers given the intervals of ∼15 nm and agreeing well with the size of single DX tiles.

We next sought to verify the assembly pathway of DNA bundles. In order to conduct this investigation, the assembly was intentionally stopped at a series of temperatures in the thermal annealing process at 60, 50, 45, 35, 30, and 25 °C, and the samples were then immediately subjected to TEM imaging (Figure 1g, Figure S5). As revealed, individual tubes began to show up at 50 °C when minimal intertube interactions should present. At 45 °C, tubes showed enhanced interactions as they began to bind and align with others. At 35 °C, loosely linked bundles of tubes were observed. Finally at 25 °C, long, wide, and compact DNA bundles with expected spatial features were formed. This experimental investigation strongly supported the hierarchical nature of the DNA bundle assembly process.

### Examination of Factors Affecting DNA Bundle Assembly

**Figure 2:**
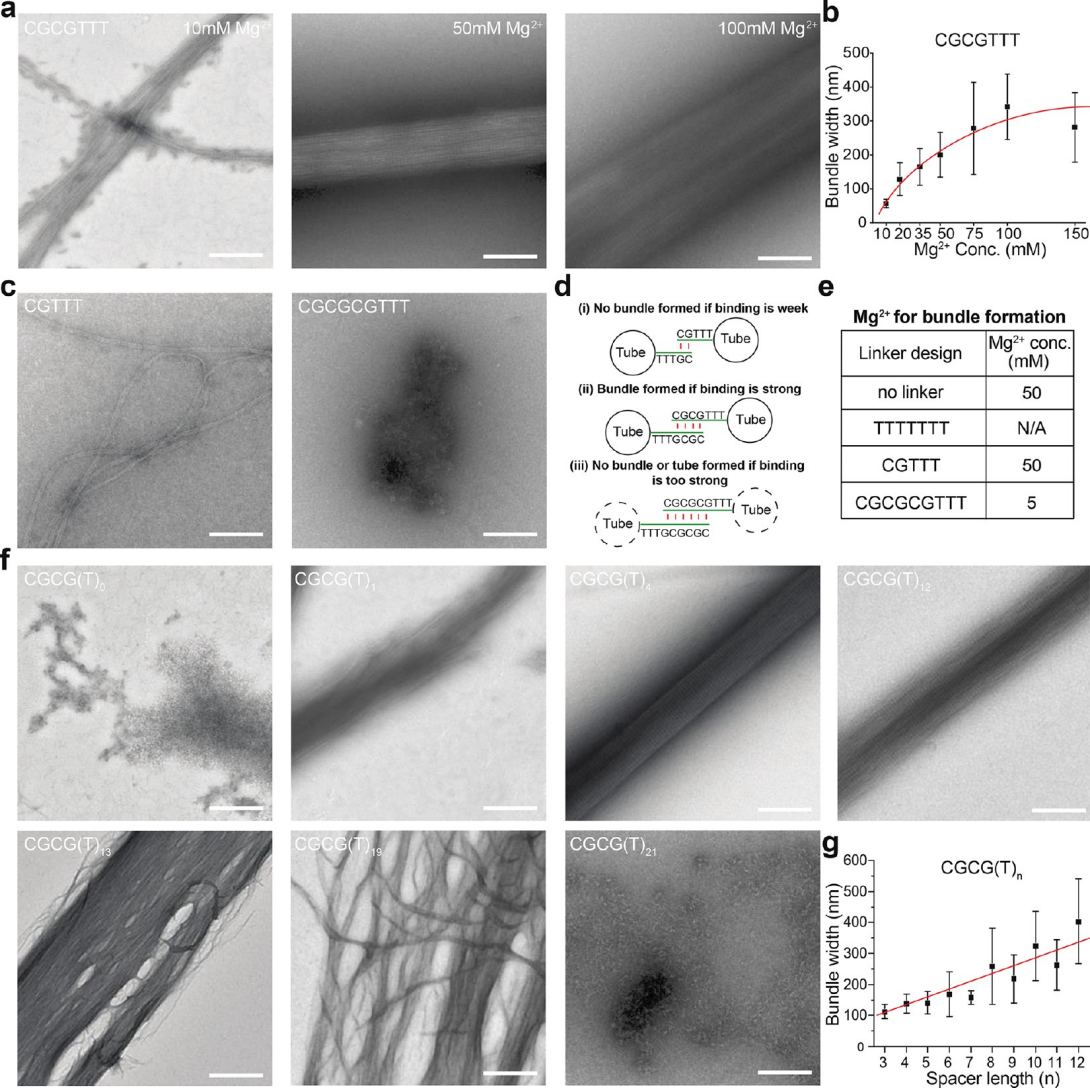
Effects of cations and linker designs on DNA bundle assembly. (a) TEM images of DNA bundles assembled at various Mg^2+^ concentrations by using the CGCGTTT linker. (b) Measured width of DNA bundles assembled at different Mg^2+^ concentrations. The measurements were conducted and averaged based on multiple bundles on TEM images. The decrease of average bundle width at 150 mM Mg^2+^, compared to 100 mM Mg^2+^, is likely due to aggregation of wider bundles at this high concentration of Mg. (c) TEM images of assembled structures with linkers of CGTTT or CGCGTTT at 20 mM Mg^2+^. For these linker designs, the binding strength was varied by tuning the number of CG on the binding domain while keeping the same spacer length. (d) Appropriate binding strength of the linker is critical to bundle formation. Weak linker leads to little intertube binding, while too strong linker disrupts tube assembly. (e) Required Mg^2+^ concentration for bundle formation varies across different linker designs. The general trend is that a linker of weaker binding strength requires a higher concentration of Mg^2+^, and vice versa. (f) TEM images of self-assembled structures with linkers of CGCG(T)_*n*_ at 20 mM Mg^2+^, where *n* ranges from 0 to 21. All linkers exhibited similar binding strengths but with varied spacer lengths. These results revealed that an appropriate spacer length (*n* = 3–12) is needed for the formation of bundles with good qualities (wide, straight, compact, and long). (g) Measured width of bundles with varied spacer length. Note: All structures were assembled from Tile A. Scale bars: 200 nm.

A comprehensive examination on how cationic strength and linker designs affect the assembly of DNA bundles was conducted. Cations (normally Mg^2+^) play pivotal roles in DNA self-assembly by shielding the negative charge of DNA backbones to facilitate DNA hybridization and self-assembly of complex DNA structures, while also promoting nonspecific or unwanted interactions between DNA strands (e.g., causing random aggregation of DNA structures). We systematically investigated a series of Mg^2+^ concentrations from 5 to 200 mM, with the linker of CGCGTTT. A general trend was that higher cationic strength led to DNA bundles of larger width (Figures 2a,b and S6a). Tubes were unable to bundle at 5 mM Mg^2+^, while specific DNA self-assembly was severely impaired at 200 mM Mg^2+^ since neither tubes nor bundles were formed. Therefore, it was revealed that an appropriate cationic strength is needed for the successful assembly of long and wide DNA bundles.

We next studied the effect of linker designs on DNA bundle assembly. First, the binding strength of DNA linkers was investigated. It was demonstrated above that a linker of CGCGTTT exhibited a potent capability to induce bundle assembly. As the binding strength of this linker design was mainly provided by C and G base pairing, for comparison, linkers of weaker (CGTTT) and stronger (CGCGCGTTT) binding strengths were designed by varying the number of CG pairs. At 20 mM Mg^2+^, a linker of CGTTT failed to provide enough intertube binding strength to induce bundle formation. Well in contrast, specific assembly of DNA tiles was disrupted if the linker (CGCGCGTTT) exhibited too strong a binding strength (Figure 2c). A linker of an appropriate binding strength was thus critical to promote the formation of DNA bundles (Figure 2d). To verify the generality of this design principle, a series of linker sequences with various binding strengths and binding patterns was designed (Figure S7) and experimentally studied (Figure S8), which revealed that there is indeed an appropriate window of binding strength for DNA bundle assembly.

For successful DNA bundle assembly, a strong correlation between appropriate binding strength of linkers and cation strength was revealed. The cohesive force of linkers and the repulsive force between tubes collectively modulate intertube interactions, with both forces largely affected by cationic strength. In principle, a higher cationic strength leads to a stronger cohesive force of DNA binding and a weaker electrostatic repulsive force. As experimentally revealed, a linker of CGTTT was able to form DNA bundles at an elevated Mg^2+^ concentration of 50 mM, while a linker of CGCGCGTTT was found capable of forming bundles at 5 mM Mg^2+^ (Figures 2e and S9a); both were unable to induce bundle assembly at a routinely used Mg^2+^ concentration of 20 mM. Similar to the report by Burns,^46^ DNA tubes with no cohesive linkers were also found able to align and bundle together at high concentrations of Mg^2+^ (50 mM, Figure S9b). In contrast, a linker of TTTTTTT remained effective at preventing intertube binding even at a high cationic strength (Figure S9c). Therefore, a linker of relatively weak binding strength is required in high cationic strength conditions, and vice versa, in order to promote DNA bundle assembly of high quality.

In addition to binding strength, the length of the T_*n*_ spacer in the linker sequence may also affect bundle assembly. We designed a set of linkers with sequences of CGCG(T)_*n*_, where *n* ranges from 0 to 21. It was revealed that long and wide DNA bundles of high quality can be assembled in the range of *n* = 3–12 (Figures 2f and S10), with a longer spacer leading to bundles of larger width that may be attributed to increased intertube spacing (Figure 2g). Defects on bundles started to show up beyond a spacer length of (T)_12_ and eventually disrupted tube and bundle assembly. Thus, we conclude that at a certain binding affinity, the spacer length of linkers shall also fall into an appropriate range to enable efficient assembly of DNA bundles. It is worth noting that for the linker with sequences of CGCGCG(T)_*n*_, a longer spacer still did not enable the assembly of bundles, suggesting the binding strength of linkers to be the predominant factor influencing bundle assembly in our experimental condition (Figure S10).

### DNA Bundles of Prescribed Features Assembled from Multiple Components

**Figure 3:**
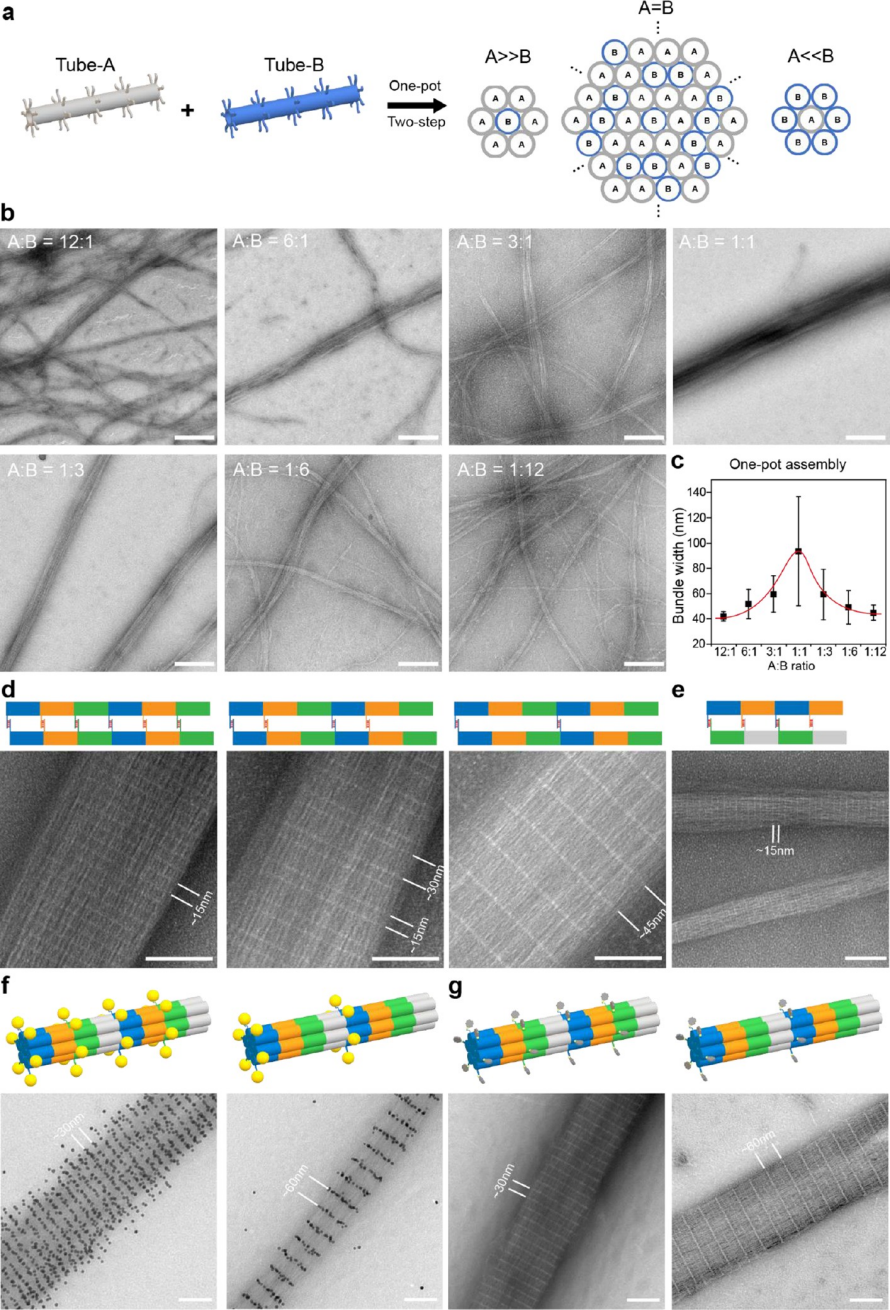
DNA bundles of prescribed features assembled from multiple distinct components. (a) Design of DNA bundles assembled from two distinct types of tubes. DNA bundles are formed via heterogeneous binding between Tube-A and Tube-B. The bundle width and composition can be modulated by adjusting the ratio between Tube-A and Tube-B. The hexagonal packing pattern illustrated here is solely for the demonstration of compact bundling. (b) Representative TEM images of DNA bundles assembled at various A:B ratios in a one-pot reaction. Scale bars: 200 nm. (c) Measured width of DNA bundles assembled in one-pot reaction. (d) Multitile DNA bundles assembled from three distinct tiles. Three types of tiles (blue, Tile C; orange, Tile D; green, Tile E) alternatively assemble into tubes first, while the homogeneous assembly of tubes led to the formation of multitile DNA bundles. Each tile contains a unique linker that can only bind to another linker from the same type of tile. The white stripes on bundles were controlled by including or omitting the linkers of designated tiles. Scale bars: 100 nm. (e) DNA bundles assembled from heterogeneous tubes composing of distinct tiles. Tile F (blue) assembles with Tile G (orange) to form tubes, while Tile H (green) assembles with Tile I (gray) to form another type of tubes. To promote bundling of tubes, linkers were designed to induce binding between Tile F-Tile H, and Tile G-Tile I. (f) Assembly of gold nanoparticles on DNA bundles. Tile J (blue) and Tile L (green) are designed to hold the capture handles for AuNP docking. Tile K (orange) and Tile M (gray) are designed to hold the cohesive linkers for DNA tube bundling. (g) Assembly of proteins on DNA bundles. The capture handles are functionalized with biotins for streptavidin (STV) binding. Note that the dimmer white stripes between the bright STV stripes are attributed to cohesive linkers. Scale bars: 100 nm.

In the above experiments, DNA bundles were assembled from DNA tiles (Tile A) of the same constitution. Here, we aim to program the structural feature of DNA bundles by designing multiple distinct types of tiles. First, we realized heterogeneous assembly of two different types of tubes with tunable bundle widths and compositions (Figure 3a). Tube-A and Tube-B were assembled from Tile A with a poly-T linker and Tile B with a poly-A linker, respectively. Given the linkers of Tube-A and Tube-B are not self-cohesive, but instead cohesive to each other, therefore, only heterogeneous bundling between Tube-A and Tube-B is allowed. By adjusting the stoichiometric ratio between two tubes, bundle width and composition may be rationally modulated (Figure 3b,c). In addition to the one-pot reaction as used above, a stepwise assembly protocol may be employed by separately assembling each type of tube first and then mixing them together with designated ratios at room temperature (Figure S11). The trend of DNA bundle width well fits with the proposed mechanism, demonstrating the successful assembly of multicomponent DNA bundles consisting of distinct types of tubes.

Next, multitile DNA bundles were fabricated (Figures 3d and S12), which were homogeneously assembled from a single type of DNA tubes consisting of three distinct tiles (Tile C, Tile D, and Tile E, Figure S1). Sticky ends on these tiles were specifically designed to enable sequential assembly of Tiles C, D, and E forming multitile DNA tubes. Furthermore, each type of tile has its own unique cohesive linker that only allows homogeneous binding to linkers on the same type of tile, e.g., the linker on Tile C can only bind to the linker on another Tile C. Multitile DNA bundles were then assembled after intertube binding was completed. By independently controlling the linkers on tiles, the white stripe features (or the intertube binding patterns) could be precisely programmed.

Finally, multicomponent DNA bundles may also be assembled by simultaneously using the above two strategies, as illustrated in Figure 3e, where two types of tubes consisting of distinct tiles heterogeneously bind to form DNA bundles. Two types of tubes were separately assembled from Tile F and Tile G, or Tile H and Tile I, respectively. Linkers on tiles were designed in such a way to enable binding between Tile F-Tile H, and Tile G-Tile I, leading to bundling of heterogeneous tubes. Similarly, the linkers may be independently modulated to fabricate DNA bundles of various spatial features (Figure S13).

To further verify the spatial features of DNA bundles and to demonstrate their utility in assembling other materials, large assemblies of gold nanoparticles (AuNPs) and streptavidin (STV) with prescribed patterns were fabricated by using DNA bundle templates. We designed a multitile DNA bundle assembled from a single type of DNA tubes that consist of four distinct tiles (Tile J, K, L, and M, Figure S1). Tile J (blue) and Tile L (green) are designed to hold the capture handles for docking guest materials. For the AuNP, the capture handles are complementary to conjugated single-strand DNA on the AuNP sequence (Table S1). For STV, the capture handles were 5′-Biotin-TTTT-3′. Tile K (orange) and Tile M (gray) are designed to have the cohesive linkers for DNA tube bundling (Figure S14). Two patterns of material assembly can be produced by including or omitting the handles on designated tiles. Two different kinds of materials, including 10 nm gold nanoparticle (AuNP, Figure 3f) and streptavidin (STV, Figure 3g), were both assembled to demonstrate the versatility of this design. TEM images revealed the successful assembly of AuNPs and STV with prescribed patterns. This result further confirmed the spatial feature of DNA bundles and showed its versatility on assembling guest materials into large structures of defined patterns

### Reconfiguration of DNA Bundles

**Figure 4:**
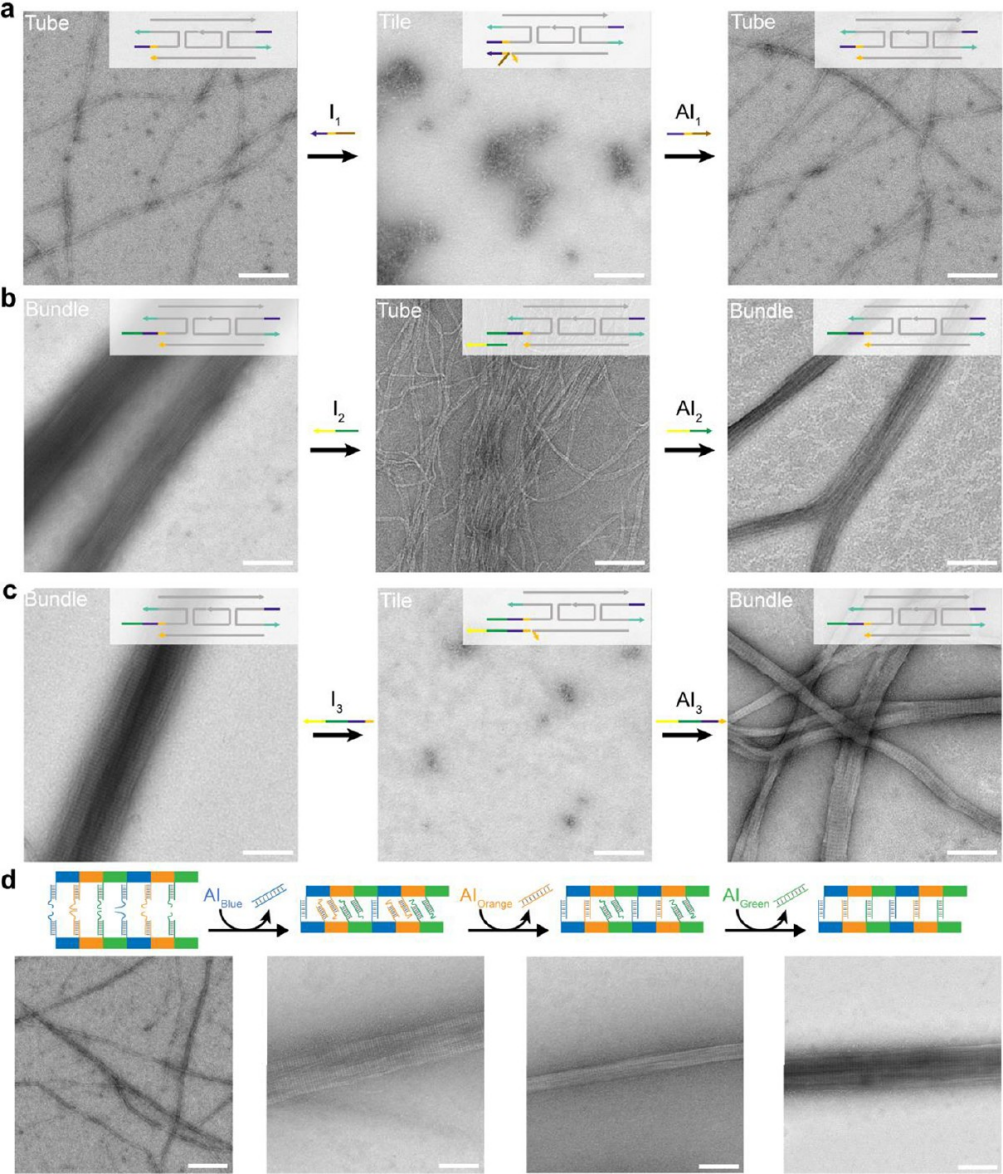
DNA bundles that reconfigure between different conformations and features. (a) Reversible reconfiguration between tubes and tiles. An inhibitor strand (I_1_) is designed to disrupt sticky-end cohesion between tiles, which can be dislocated from the tile by the anti-inhibitor strand (AI_1_) via a strand displacement reaction. (b) Reversible reconfiguration between bundles and tubes. An inhibitor strand (I_2_) is designed to disrupt the linker binding between tubes, whose disruptive effect can be reversed by the anti-inhibitor strand (AI_2_). (c) Reversible reconfiguration between bundles and tiles. An inhibitor strand (I_3_) is designed to disrupt both sticky-end cohesion and linker binding, whose deconstructive effect can be neutralized by the anti-inhibitor strand (AI_3_). Note: Tile A was used for these above experiments. (d) Dynamic transformation of tubes to bundles with tunable features. Tile C (blue), Tile D (orange), and Tile E (green) were alternatively assembled into tubes with their linkers being inhibited. Each tile contains a unique linker that can only bind to another linker from the same type of tile. Linkers were sequentially activated to induce intertube binding for the formation of multitile DNA bundles with tunable features and intertube binding patterns. Scale bars: 200 nm.

Dynamic reconfiguration of tubular nanostructures has been realized to fulfill various applications.^29,31,48,49^ Herein, we sought to integrate dynamic reconfigurability to our large DNA bundle structures that may further enrich the toolbox of dynamic DNA systems. The reconfigurability was implemented by using toehold-mediated strand displacement reactions to inhibit/activate binding between linkers (for bundle assembly) and/or between sticky-ends (for tube assembly). Using this strategy, three types of reversible transformations were demonstrated, which are among tube and tile, bundle and tube, or bundle and tile. Agarose gel electrophoresis was first used to verify the transformation reactions. Motilities of corresponding bands suggested the successful reconfigurations between DNA structures (Figure S15). TEM was then used to directly visualize the structures before and after reconfigurations (Figure 4a–c). It was revealed that all three types of reconfigurations can be readily executed in a reversible manner. Restored bundles had an average width of 91.3 nm (from tube, Figure 4b) or 80.2 nm (from tile, Figure 4c), slightly narrower than initially one-pot-assembled bundles having an average width of 112.0 nm. We believe this difference is due to the fact that the reconfiguration and restoration of DNA bundles was carried out under room temperature that was less efficient than the thermal annealing (18 h) for bundle assembly.

Furthermore, we implemented the dynamic capability of multicomponent multitile DNA bundle design by sequentially activating linkers to strengthen intertube binding forces in a stepwise manner (Figures 4d and S16). All three distinct cohesive linkers on DNA tiles (Tile C, Tile D, and Tile E) were initially inhibited by inhibitors, thus only tubes were produced in the first place. Activation of the blue linkers (Tile C) led to the formation of loose bundles of observable defects given relatively weak intertube binding was provided by one type of linkers. Further activation of the orange (Tile D) and green (Tile E) linkers induced stronger intertube binding forces that started to yield more compact and wide DNA bundles.

## Conclusion

In this report, we developed a simple yet versatile molecular assembling strategy for the rational fabrication of long (dozens of micrometers) and wide (hundreds of nanometers) DNA bundle structures of defined spatial features and dynamic capabilities. The basic building block of this self-assembly system is the DNA DX tile with a cohesive linker. DNA bundles were assembled following a hierarchical pathway, where DNA tiles first assembled into tubes and then into bundles via linker-induced intertube binding. Comprehensive studies revealed that the formation of DNA bundles was collectively determined by a number of factors including linker designs (binding strength, spacer length, linker position, etc.) and cationic strength. Furthermore, we developed a number of assembling strategies to produce a variety of multicomponent DNA bundles of controllable spatial features and compositions by designing distinct types of tiles for programmable tile assembly and intertube binding. These multicomponent DNA bundles were further employed in the fabrication of large AuNP and STV structures with prescribed patterns as determined by the bundle templates. Finally, DNA bundles exhibited robust and rationally controllable reconfigurability following specific molecular stimulations that can transform to/from tiles or tubes.

The current assembling system exhibits several noteworthy advantages in terms of fabricating DNA bundle structures with such large dimensions and versatile functionalities. First, it is simple and cost-effective, given that only five short DNA strands are needed for DNA bundle assembly. Previously reported large one-dimensional structures of comparable dimensions and controllability were mostly assembled from DNA origami or DNA brick units that is complex and expensive.^12,13,26^ The work by Burns employed high concentrations of cations to condense tubes into bundles, which lacks rational control over bundle features, and a high cationic strength may limit the subsequent applications of bundles. Second, DNA bundles of prescribed features may be readily achieved via inducing slight changes into DNA tile designs, which is simple, robust, and versatile. Third, our DNA bundles exhibit a versatile dynamic capability that can readily reconfigure among various conformations by using specific molecular triggers. With their large sizes and capability of reversible transformations, the DNA bundles reported in this work hold great application potential in various fields. For example, they may serve as biomimetic cellular skeletons for constructing artificial cells or organelles for synthetic biology studies. Or they may be used as templates to direct the assembly of functional materials forming macroscopic materials with emerging properties. They may also find utility in building artificial tissues such as muscle fibers.

## Methods

### DNA Strand Preparation

All DNA strands involved in this paper were directly purchased either from Integrated DNA Technologies (IDT) or from Sangon Biotech (Shanghai) Co., Ltd. Strands ordered from these two companies were tested to show minimal difference in tests. For strands longer than 60 nt, PAGE purification was ordered from the company. All strands were dissolved in deionized water to a final concentration of 100 uM for later use.

### DNA Sequence Design

There are 3 parts in one DX tile: Backbone, sticky ends, and linker. Backbone sequences are designed using Uniquimer.^1^ Sticky ends are generated with random sequences. Linkers are designed according to the rules described in the paper. Their orthogonality was tested using NUPACK.^2^

### DX Tile Backbone Used for All Experiments

All DX tile backbone sequences are shown in Figure S1. For specific experiments, the DX tile backbone used is indicated in the main text and figures. Otherwise, all other experiments used Tile A.

### NUPACK and Oligoanalyzer Simulation

The simulation on NUPACK was performed using the analysis tool on the Web site. Parameters were chosen to meet the actual reaction condition. This simulation is used to check the binding pattern of linkers and the orthogonality of DX tile strands. The simulation on oligoanalyzer was performed referencing to the guidance posted on the IDT Web site. Parameters were chosen to meet the actual reaction condition. This simulation is used to check the binding pattern of linkers and their binding delta G value.

### Annealing Process

The assembly for all trials was carried out in 1× TE buffer with a corresponding concentration of MgCl_2_. The annealing process to assemble DX tiles, most nanotubes, and bundles was this protocol: (1) 85 °C for 10 min, (2) lower the temperature to 65 °C, then slowly decrease temperature (−0.1 °C for every 10 min) from 65 to 25 °C. For specific trials, like the investigation of nanotube and bundle formation under different temperatures, modifications were made accordingly. The annealing was carried out using Bio Rad C1000 Touch Thermal Cyclers.

### One-Pot and Two-Step Assembly of A-B Bundles

For one-pot assembly, both DX tiles for Tube-A and Tube-B were mixed together in one tube and underwent the annealing process above-mentioned. For 2-step assembly, DX tiles for Tube-A and Tube-B were annealed in different tubes, respectively. After annealing, the products were mixed into one tube and shaken under room temperature overnight.

### Gold Nanoparticle (AuNP) and Streptavidin (STV) Conjugation on DNA Bundles

Here, the 10 nm AuNP was ordered from Ted Pella. STV was ordered from New England Biolabs. The DNA bundle consisting of Tile J, K, L, and M with the corresponding cohesive linker and handle sequence was first annealed with a normal annealing protocol. The annealed DNA bundle was then mixed with payloads and shaken under room temperature for 2 h. For AuNP conjugation, the functionalization of the AuNP with Thiol-labeled single-strand DNA was carried out as in the previous study.^3^ The thiol-DNA was first reduced with enough TCEP and then mixed with the 10 nm AuNP in a 300:1 ratio. The mixture was frozen in −20 °C overnight, then thawed under room temperature for 1 h. The solution was then centrifuged at 12000*g* for 20 min, and then the supernatant was removed. The AuNP pellet was washed with water twice, and additional centrifuge steps were used to remove water after each washing. The recovered AuNP was dissolved in a 1× TE 20 mM Mg^2+^ buffer and used at the ratio of 1:10 to DX tiles. For STV conjugation, STV was diluted into a 1× TE 20 mM Mg^2+^ buffer and used at the ratio of 1:5 to DX tiles.

### Dynamic Control of the Assembly Process

For all invader and anti-invader reactions, two kinds of methods were tested. The first method was to mix the DX tile and the invader/anti-invader together, which then underwent the annealing process. The second method was to anneal the starting structure (tile, tube, or bundle) first. Then, the invader/anti-invader was added and the tube was shaken at room or the corresponding temperature overnight. The concentration of invader added was 2 times that of DX tile’s, and the concentration of anti-invader added was 2 times that of invader’s.

### Transmission Electron Microscopy (TEM) Imaging

For the sample preparation for TEM imaging, a 3 μL sample was deposited on the surface charged carbon film coated copper EM grids for 30 s. Then, a filter paper was used to remove the excess liquid on the grid. Next, 8 μL of a 1% uranyl formate (UF) solution was used for negative staining, and excess liquid was also removed with a filter paper after 20 s. The samples were imaged using a Hitachi HT-7700 120 kV W (Tungsten) TEM with an AMT CCD camera. Note that the number of nanostructures shown on the images does not represent the actual yield of them, and the brightness of the figures solely depends on the staining and imaging technique. To prepare the 1% UF solution, 10 mg of UF powder was dissolved in 1 mL of DI water and heated to the point when no changes further appeared. Then, 1 μL of 5 M NaOH was added to the solution and mixed well. The solution was further filtered using a 0.2 μm syringe filter with a cellulose acetate membrane, and the filtrate was collected. Copper EM grids were charged using a Pelco easiGlow Glow Discharge Cleaning System. The UF powder was purchased from Electron Microscopy Sciences. The 5 M NaOH was purchased from Fisher Scientific. EM grids were purchased from Electron Microscopy Sciences, and the model of grid was CF400-CU. The 0.2 μm syringe filter was purchased from VWR International.

### Agarose Gel Electrophoresis

A nondenaturing agarose gel was prepared according to the standard protocol provided by Thermo Scientific. A 1% agarose gel was prepared with 0.5× TBE and 10 mM Mg^2+^ for the separation of DX tiles and assembled nanotubes and bundles. A 100 ng sample was loaded in each well. The electrophoresis was carried out at 60 V for 90 min, and ethidium bromide was used for staining. The gel was imaged using a Bio-Rad Gel Doc EZ Imager.

### Confocal Microscopy Imaging

Confocal images were obtained by confocal microscopy (Leica TCS SP8) with 63×/1.40 NA oil immersion objectives. For sample preparation, a 5 μL droplet (200 nM for bundles and 20 nM for tubes) of sample was pipetted onto a glass slide and then covered by a coverslip. Tubes with fluorophores were imaged using a corresponding laser exciter and filter.

### Measuring the Size of Nanostructures Using ImageJ

The size of the structures in TEM images was analyzed using ImageJ and plotted to exhibit the trend. A TEM image is evenly divided into 9 squares (3by3), and the width of the bundle near the center of each square was measured. At least 25 data points were randomly selected for each group, and their average value was calculated and plotted. See Figure S6 for more details.

## Supporting Information Available

DNA strand sequence, additional TEM images, statistical data of DNA bundle property measurement, and detailed schematics of designs in Figure 4 (PDF)
