# Supplementary material for: Building Large DNA Bundles via Controlled Hierarchical Assembly of DNA Tubes
Source: ACS Nano. 2023 May 19;17(11):10486–95. doi: 10.1021/acsnano.3c01342 (PMC10278166; doi:10.1021/acsnano.3c01342)
Supplement: Supplementary file 1 — nn3c01342_si_001.pdf [file nn3c01342_si_001.pdf]

Building Large DNA Bundles via Controlled Hierarchical Assembly of DNA Tubes

Yunlong Zhang[1], Donglei Yang[2*], Pengfei Wang[2*], Yonggang Ke[1,3*]

[1]Department of Chemistry, Emory University, Atlanta, Georgia 30322, USA

[2]Institute of Molecular Medicine, Department of Laboratory Medicine, Shanghai Key Laboratory for Nucleic Acid Chemistry and Nanomedicine, Center for DNA Information Storage, Renji Hospital, School of Medicine, Shanghai Jiao Tong University, Shanghai 200127, China

[3]Wallace H. Coulter Department of Biomedical Engineering, Georgia Institute of Technology and Emory University, Atlanta, Georgia 30322, USA

[#] These authors contributed equally

*Email: yonggang.ke@emory.edu, pengfei.wang@sjtu.edu.cn, dongleiyang@shsmu.edu.cn

Supplemental figures

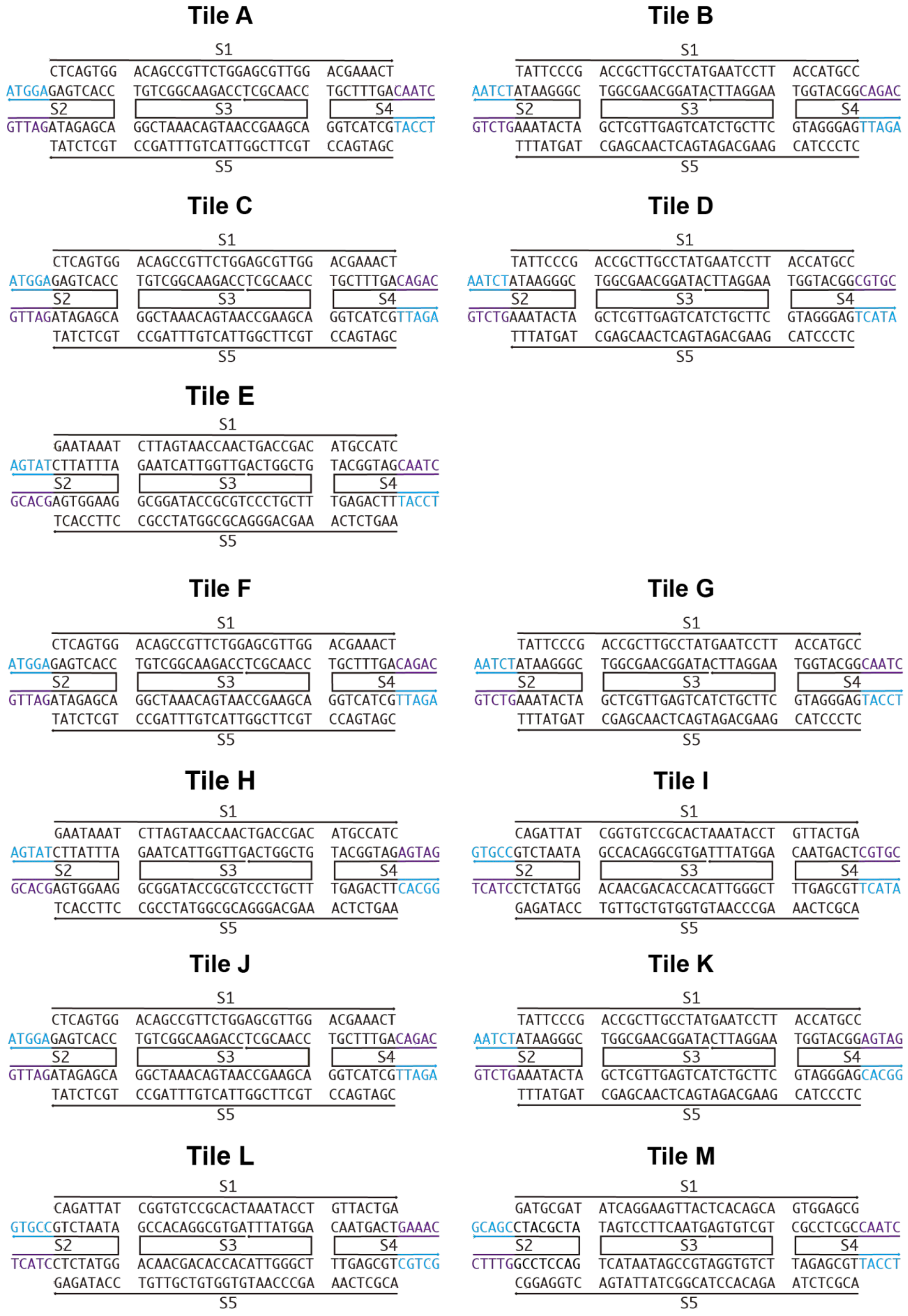

Figure S1. Designs and sequence of all DNA tiles used in this paper. Linker sequences are extended from the 5' end of S2 strand unless specified. Some tiles share the same backbone, like Tile A and C.

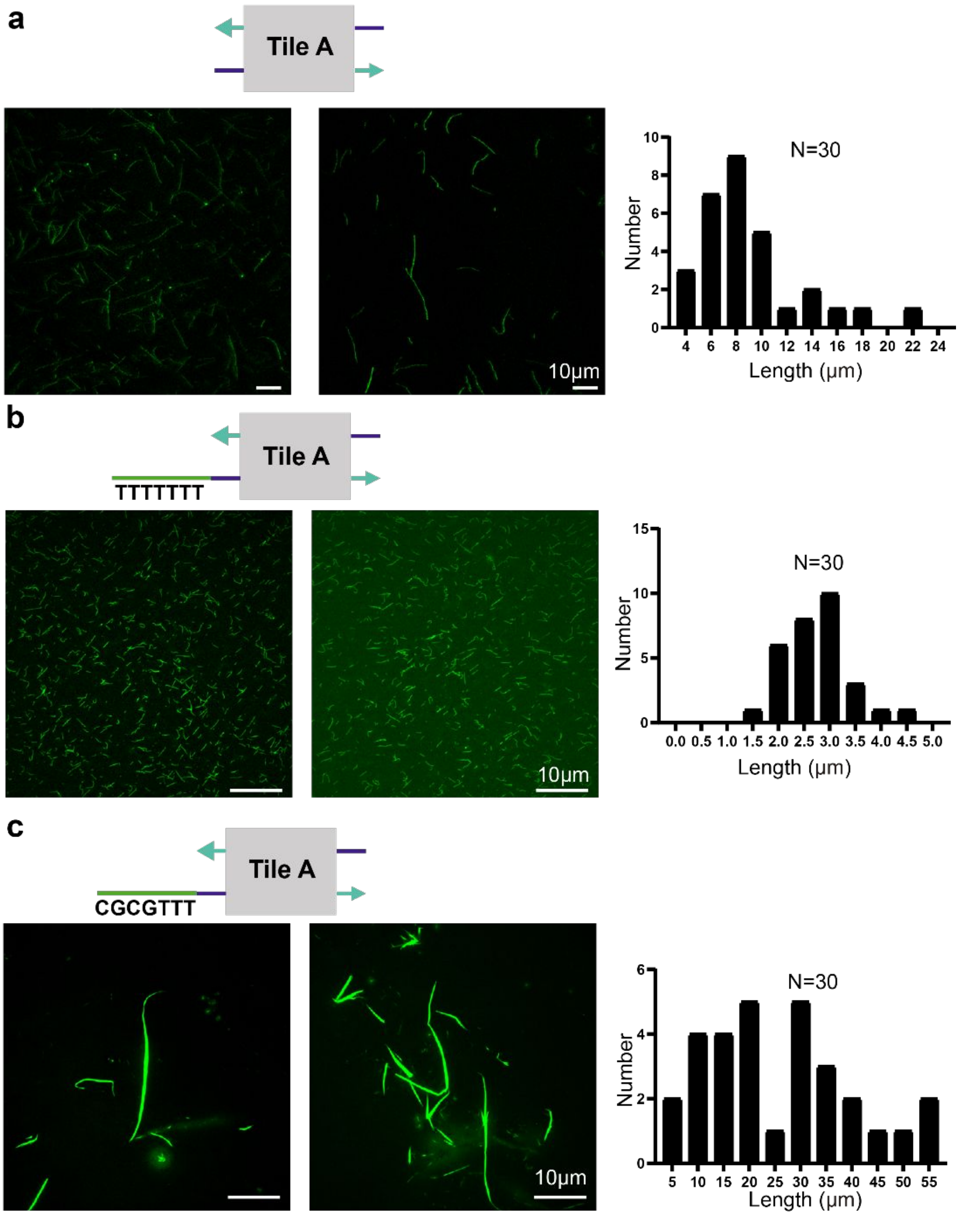

Figure S2. Representative CLSM images for DNA tubes and DNA bundles. (a) DNA tubes assembled from Tile A with no linker. (b) DNA tubes assembled from Tile A with a non-cohesive linker of TTTTTTT. (c) DNA bundles assembled from Tile A with a cohesive linker of CGCGTTT.

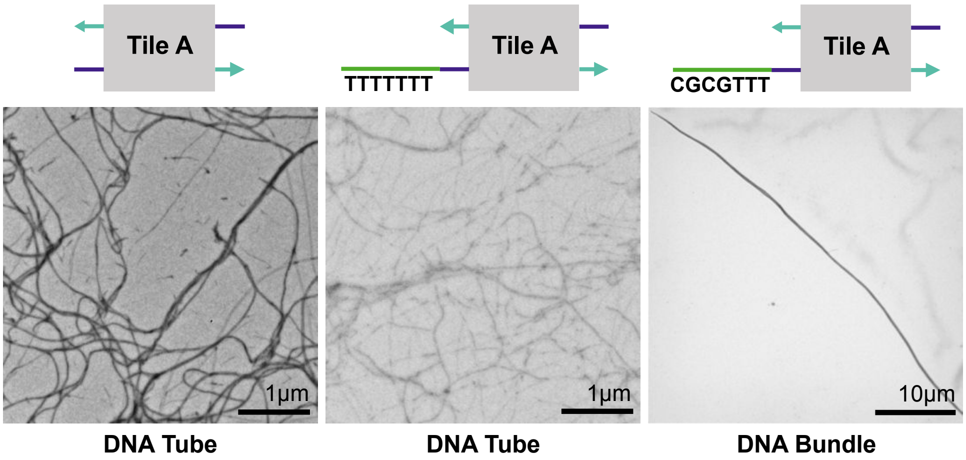

Figure S3. Representative TEM images of DNA tubes and DNA bundles assembled from Tile A with designated linker sequences.

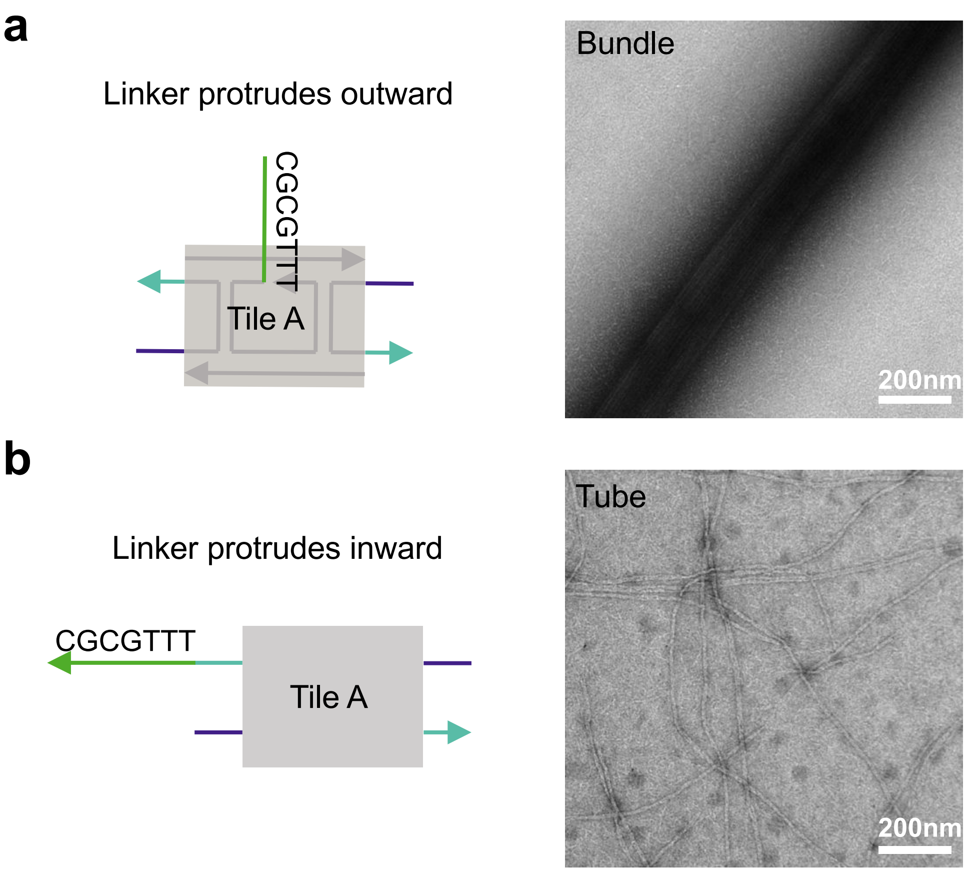

Figure S4. Linker position affects DNA bundle assembly. (a) DNA bundles were formed if linker is positioned on the 5' end of S3 strand that protruding outward from tube outer surface. (b) DNA tubes were formed if linker is positioned on the 3' end of S2 strand that protruding inward towards the tube tunnel. Please refer to the previous work of using the similar DX tile design for the method to control and confirm the chirality and linker orientation when forming nanotubes.[4, 5]

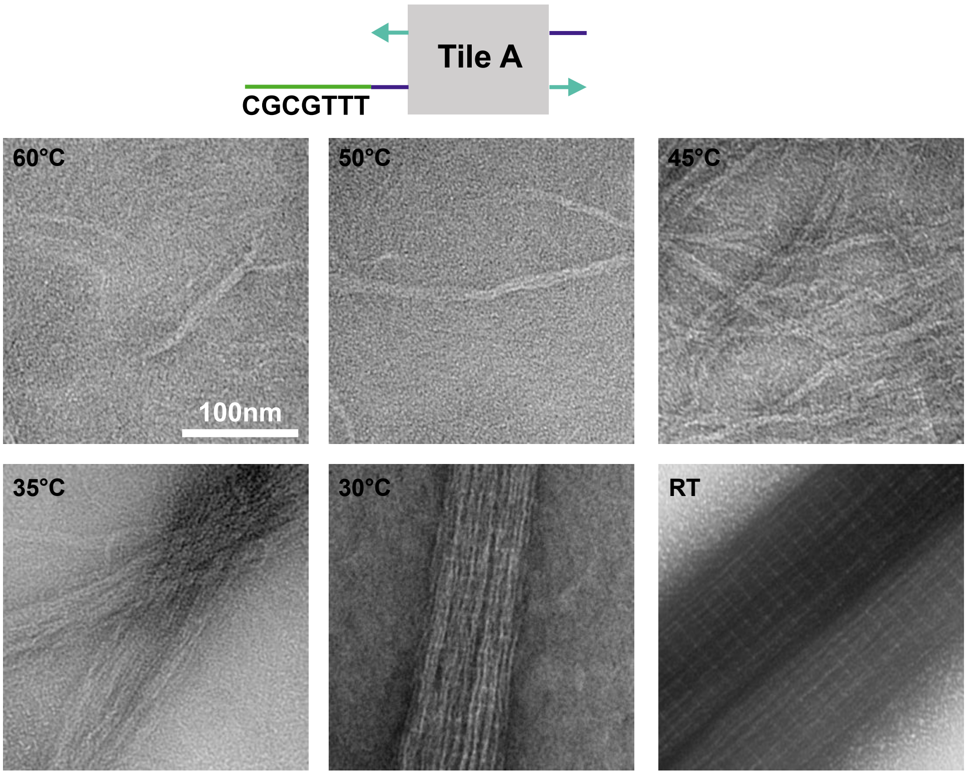

Figure S5. DNA bundles are hierarchically assembled. The DNA tiles are heated to 85°C, then gradually cooled down to designated temperatures for characterization. All images share the same scale bar.

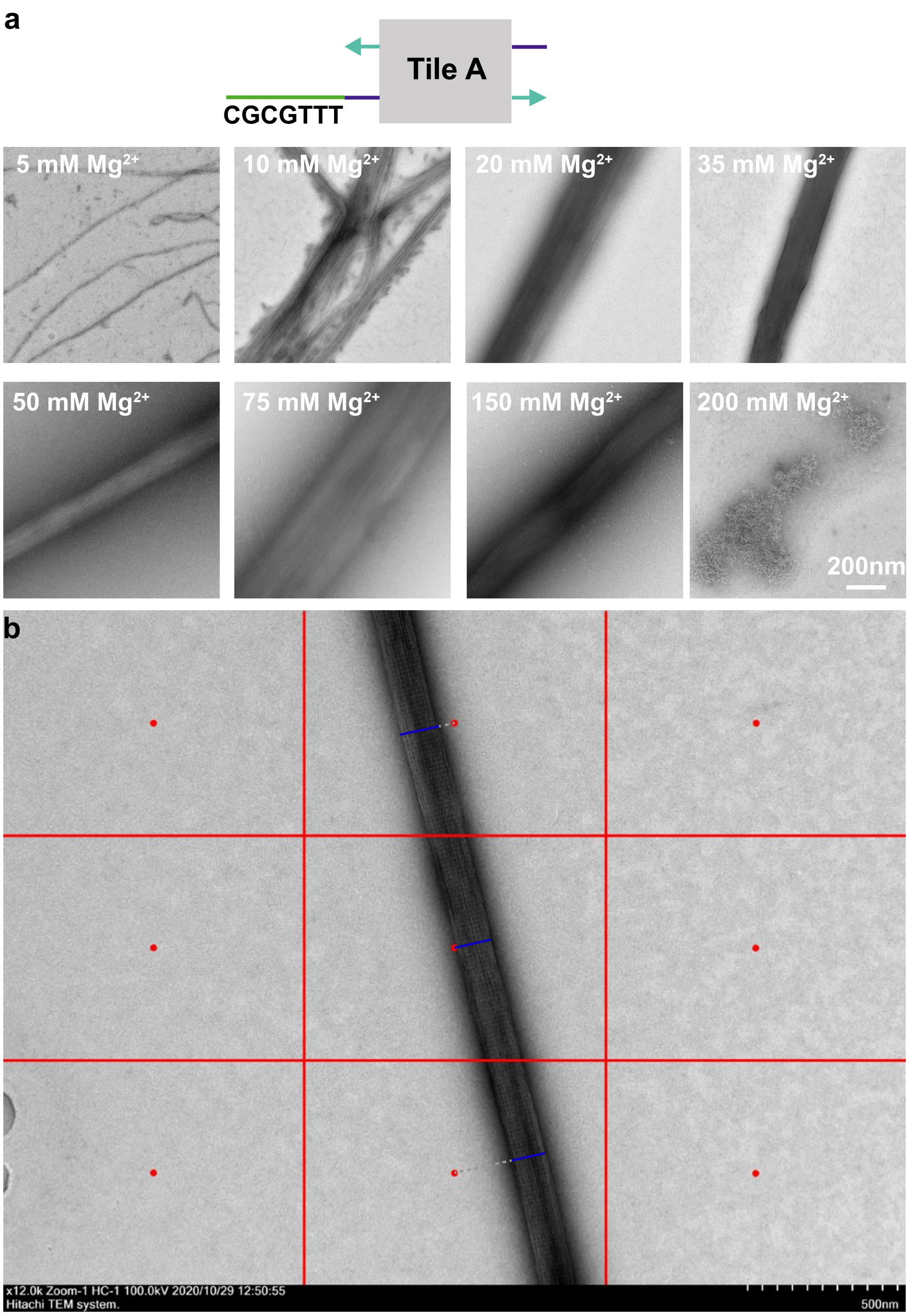

Figure S6. Cationic strength plays pivotal role in affecting DNA bundle assembly. (a) There is an appropriate window for cationic strength to promote DNA bundle formation. In the case of Tile A with linker of CGCGTTT, the Mg^{2+} concentration shall fall within the window of 10-150 mM to induce DNA bundle assembly. All figures share the same scale bar: 200 nm. (b) Measurement of bundle width using ImageJ. The original TEM image is evenly divided into 9 segments (Indicated by red lines). The center of each segment is labeled using red dots. In each segment, the bundle segment which is nearest to the center red dot (Indicated by grey dashed lines) is selected, and the width of selected bundle segment is measured (Indicated by blue lines). For each experimental group, at least 25 data points are collected through out multiple TEM images.

Forms bundle?	Sequence 5’-3’	Base pair number	Binding Pattern	NUPACK Simulation
Yes	ATCTGCA	4bp	ATCTGCA<br>ATCTGCA	Delta G = -7.11kcal/mol
	CGCAGCA	4bp	CGCAGCA<br>CGCAGCA	Delta G = -8.10kcal/mol
	CGCGTTT	4bp	CGCGTTT<br>CGCGTTT	Delta G = -9.30kcal/mol
	TCGTCGT	4bp	TCGTCGT<br>TCGTCGT	Delta G = -7.56kcal/mol
	TTTCGCG	4bp	TTTCGCG<br>TTTCGCG	Delta G = -9.69kcal/mol
	AAAAAAA<br>+<br>TTTTTTT	7bp	AAAAAAA<br>TTTTTTT	Delta G = -8.70kcal/mol
No	ACGTACGT	8bp	ACGTACGT<br>ACGTACGT	Delta G = -14.09kcal/mol
	CGCGCGT	6bp	CGCGCGT<br>CGCGCGT	Delta G = -14.67kcal/mol
	GCTAACT	4bp	GCTAACT<br>GCTAACT	No self-binding
	ATCTACA	4bp	ATCTACA<br>ATCTACA	No self-binding
	TGGTATT	2bp	TGGTATT<br>TGGTATT	No self-binding
	CGTTTTT	2bp	CGTTTTT<br>CGTTTTT	No self-binding
	TTTTTCG	2bp	TTTTTCG<br>TTTTTCG	No self-binding
	TTTTTTT	0bp	N/A	No self-binding

Figure S7. All linker sequences designed and studied. Simulations are done under the condition with 100 mM of Na^+ and 20 mM of Mg^{2+}. By comparing the NUPACK simulation result, the conclusion can be drawn that the self-binding strength (delta G) of a linker should be in a certain range to enable the assembly of DNA bundle structure with certain Mg^{2+} concentration.

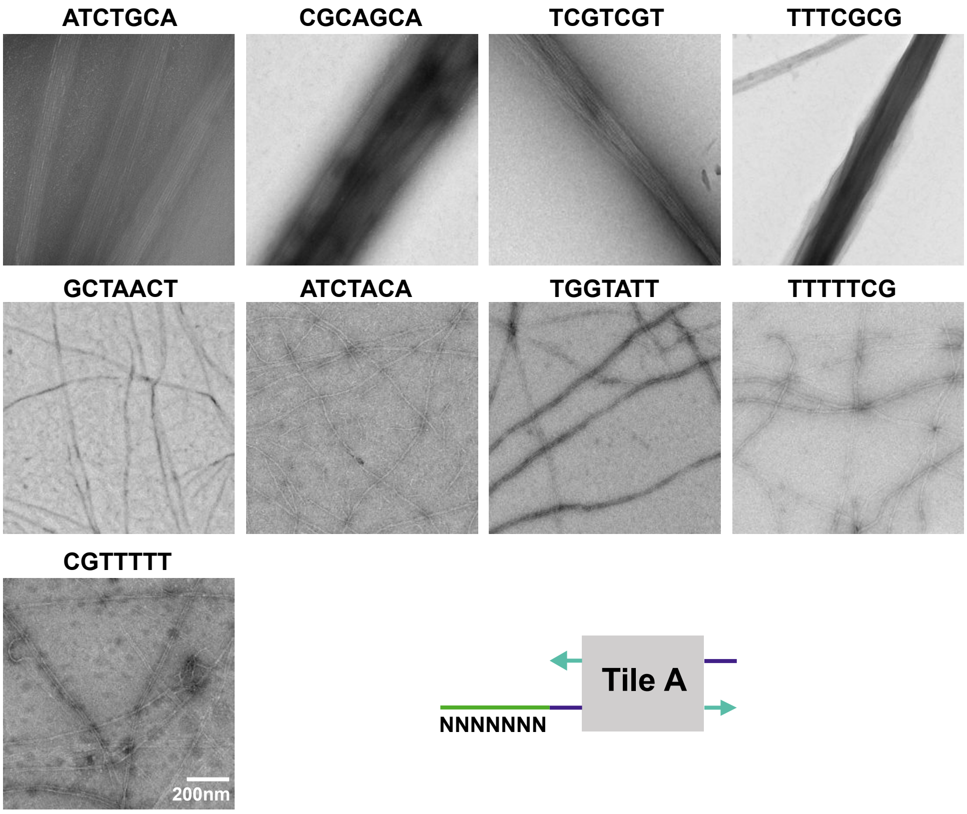

Figure S8. TEM images of assembly results for some linker designs in Figure S7. The designs included in other figures are not presented here, and the designs that does not yield DNA tubes or bundles are not included. All images share the same scale bar of 200 nm.

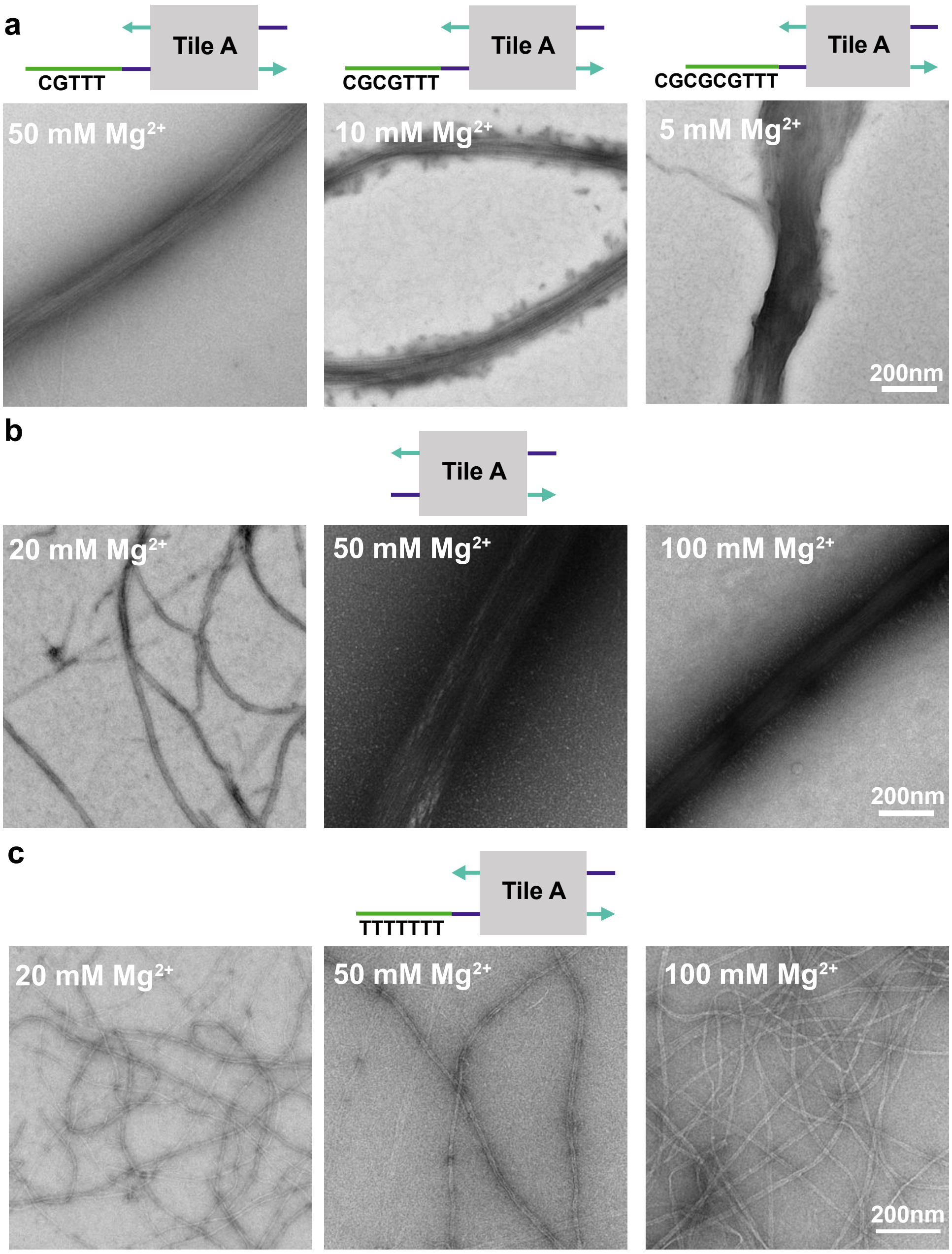

Figure S9. Cationic concentration needed for DNA bundle assembly is highly dependent on the binding strength of linkers. (a) Higher cationic strength is needed for weaker linkers, while lower cationic concentration is demanded by stronger linkers. (b) DNA tile with no linker can even form DNA bundles at sufficiently high concentrations of Mg^{2+}. (c) DNA tile with a non-cohesive linker of TTTTTTT is unable to form DNA bundles even at 100 mM of Mg^{2+}. All figures share the same scale bar of 200 nm.

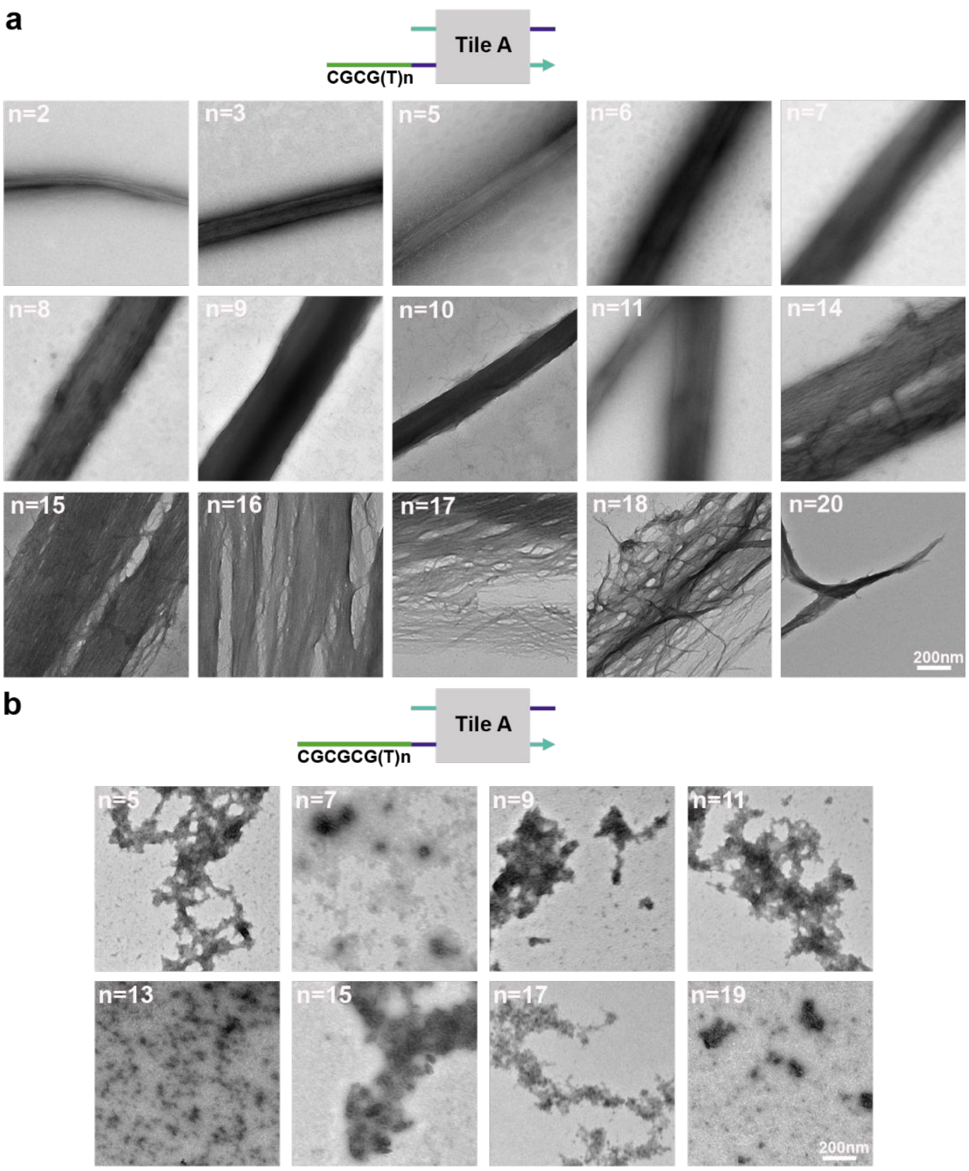

Figure S10. The spacer length on linkers also influences DNA bundle assembly. (a) The linker sequences used here are CGCG$(T)_n$, where n varies from 0-21. (b) The linker sequences used here are CGCGCG$(T)_n$, where n varies from 5-19. All groups are assembled with 20 mM of Mg^{2+}. All figures share the same scale bar.

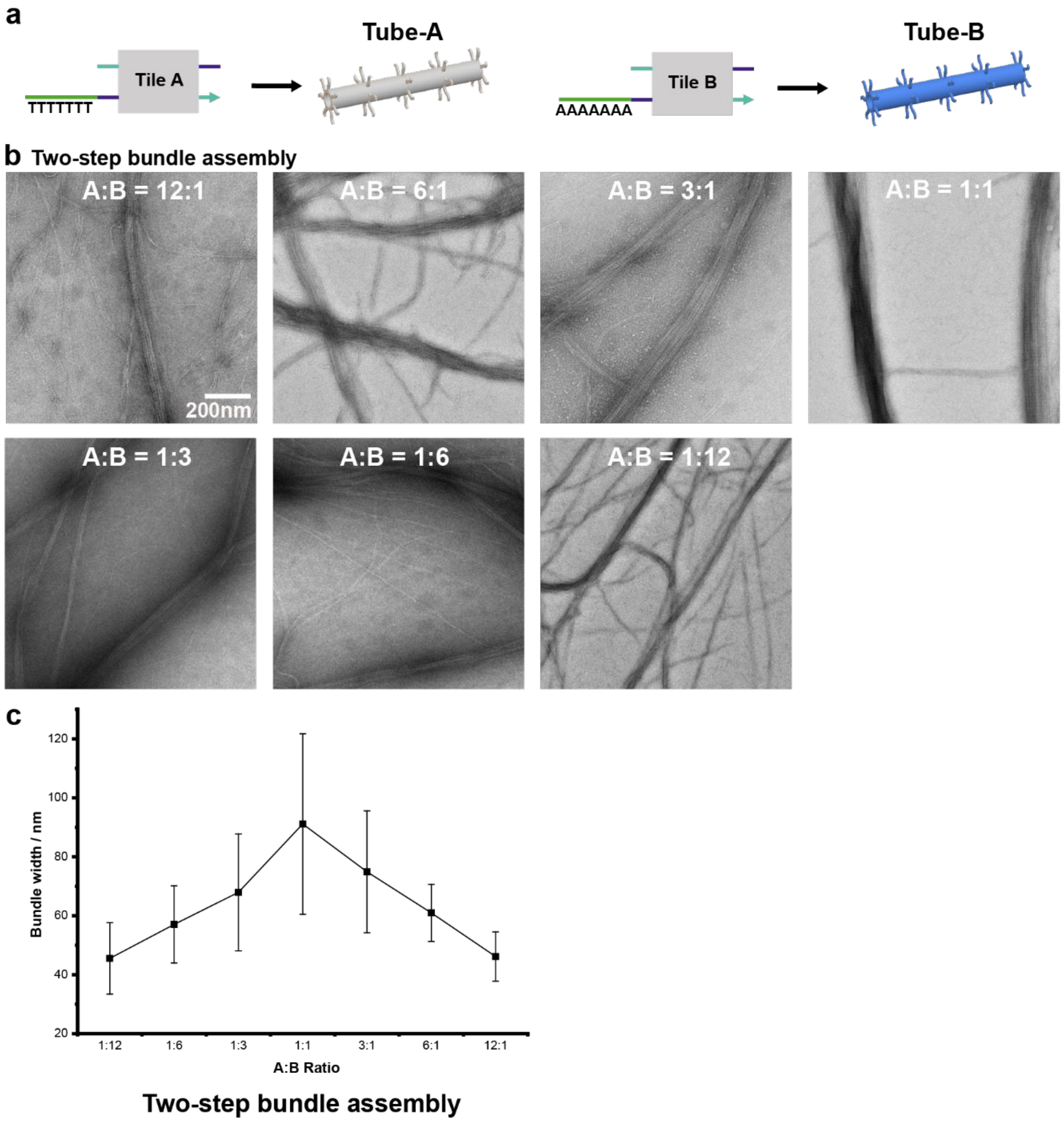

Figure S11. Multicomponent DNA bundles assembled from Tube-A and Tube-B. (a) Schematics for tube assembly. (b) Representative TEM images of DNA bundles assembled with different ratios of Tube-A and Tube-B. All images share the same scale bar of 200 nm. (c) The average width of DNA bundles at different ratios.

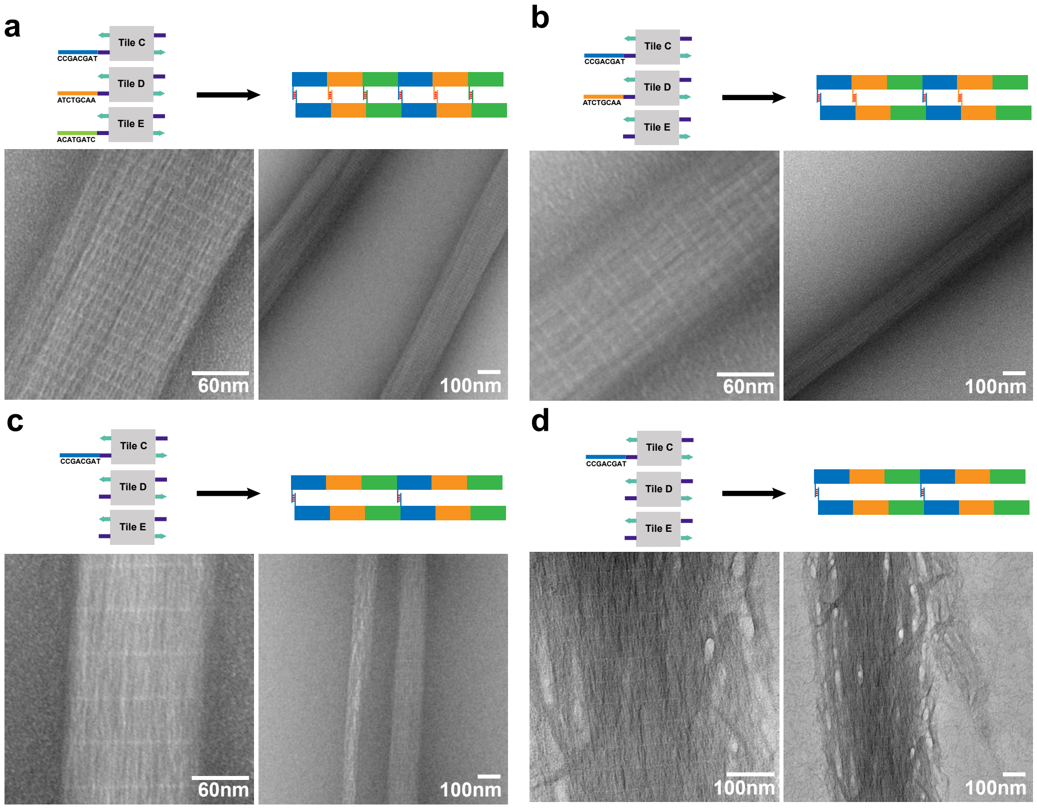

Figure S12. Multi-tile DNA bundles. (a-c) DNA bundles assembled from three distinct tiles. Each tile has a unique linker that can only bind to the linker on another same type of tile. The presence of linkers may be controlled to program the spatial features (white stripe patterns) on DNA bundles. In this experiment, DNA bundles were assembled with 40 mM of Mg^{2+}. (d) DNA bundles cannot be well formed at 20 mM of Mg^{2+} if only Tile C having the linker given the binding strength may not be sufficient at this low cationic strength.

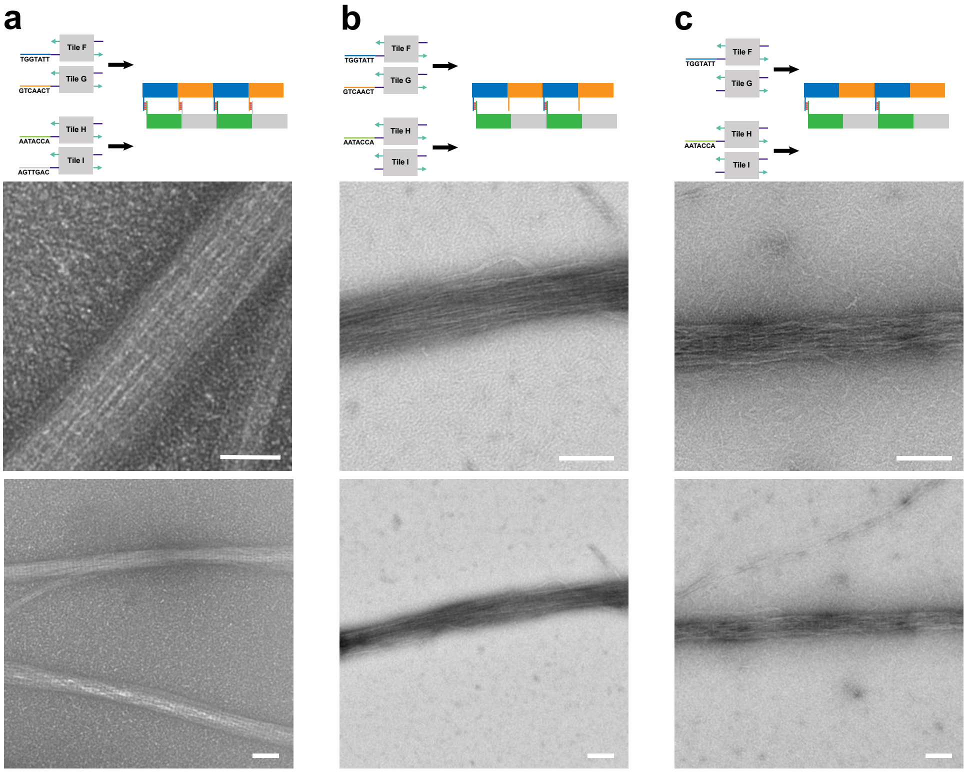

d

Bundle type	a	b	c
Average width / nm	73.2	57.4	51.6
Interval / nm	15	15	30

Figure S13. Multicomponent DNA bundles assembled from distinct tubes consisting of distinct tiles. (a-c) Assembling schemes and TEM images for DNA bundles. Scale bars: 100 nm. (d) Morphology data of DNA bundles in three experimental groups. Lower number of linkers led to DNA bundles of smaller width. Besides, the white stripe features agree well to the designs.

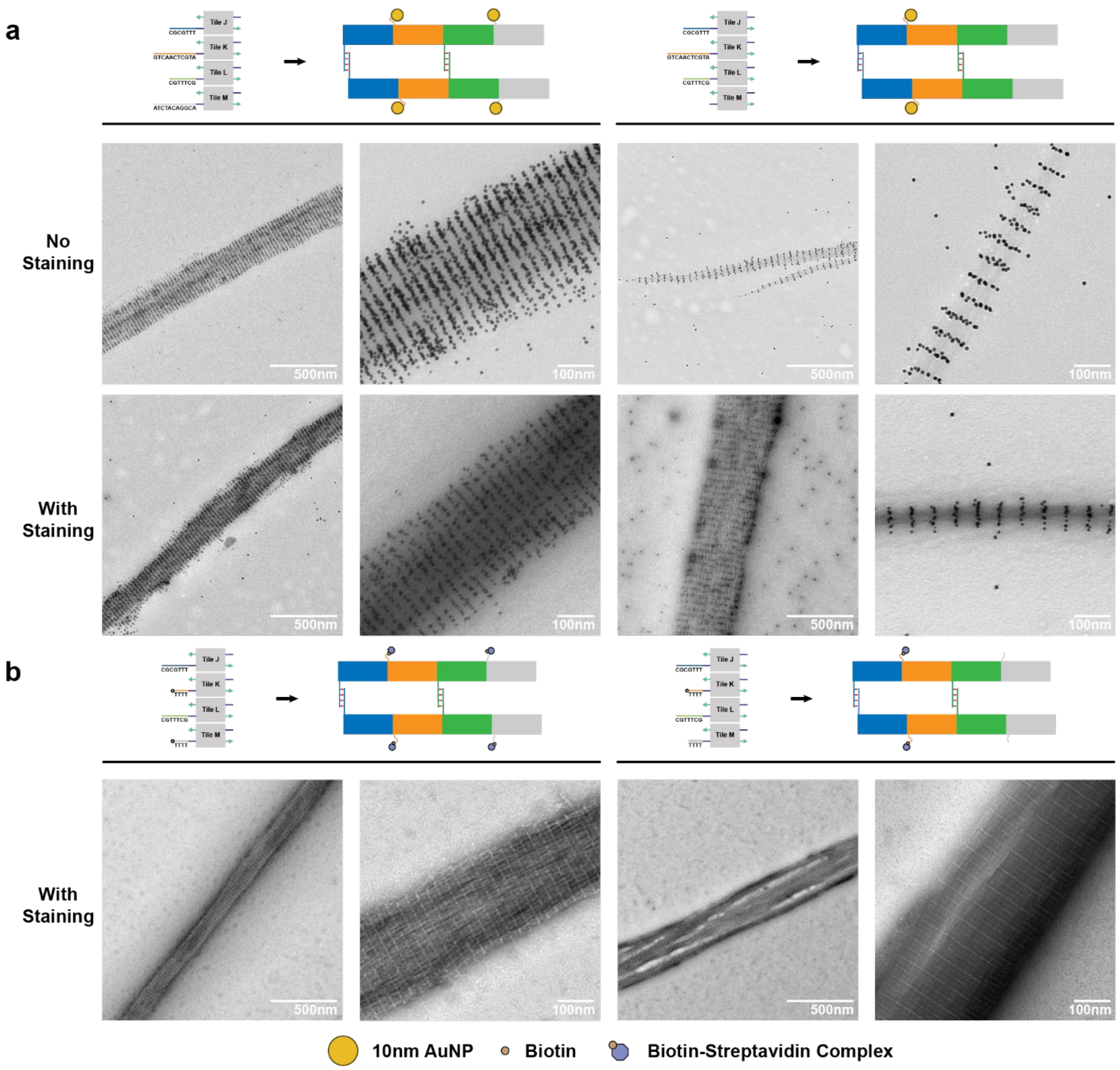

Figure S14. AuNP and STV conjugation on DNA bundles. (**a**) 10nm AuNP conjugation on DNA bundles. The composition of the assembled DNA bundle is illustrated, and representative TEM images, either stained or not stained with 1% Uranium formate, are presented for the design with 2 AuNP per cycle and the design with 1 AuNP per cycle. The stained sample shows a darker image of DNA bundles bearing AuNPs. (**b**) STV conjugation on DNA bundles. The composition of the assembled DNA bundle is illustrated, and representative TEM images are presented for the design with 2 STV per cycle and the design with 1 STV per cycle. For the details of the design and assembly process, please refer to main figure 3 and Material and Method section.

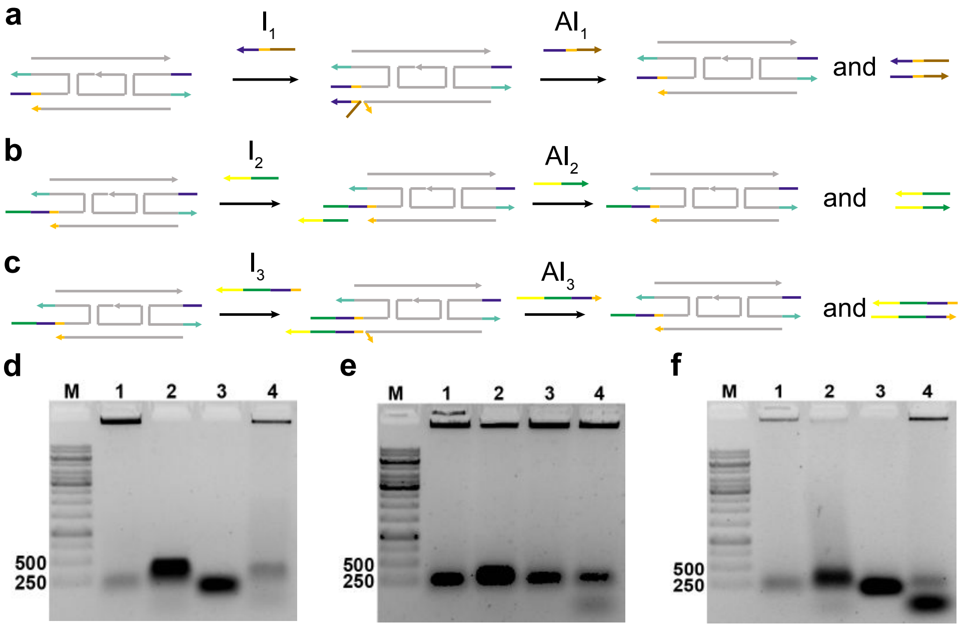

Figure S15. Schematics and gel electrophoresis for DNA bundle reconfigurations. (a, d) Reversible reconfigurations between DNA tubes and DNA tiles. (b, e) Reversible reconfigurations between DNA bundles and DNA tubes. (c, f) Reversible reconfigurations between DNA bundles and DNA tiles. All gels share the same sample setup among all 5 lanes. M: DNA marker. Lane 1: Original assembly. Lane 2: Pre-annealed original assembly mixed with inhibitors and incubated under RT. Lane 3: original assembly annealed together with inhibitors. Lane 4: Lane 3's product mixed with anti-inhibitors and incubated under RT.

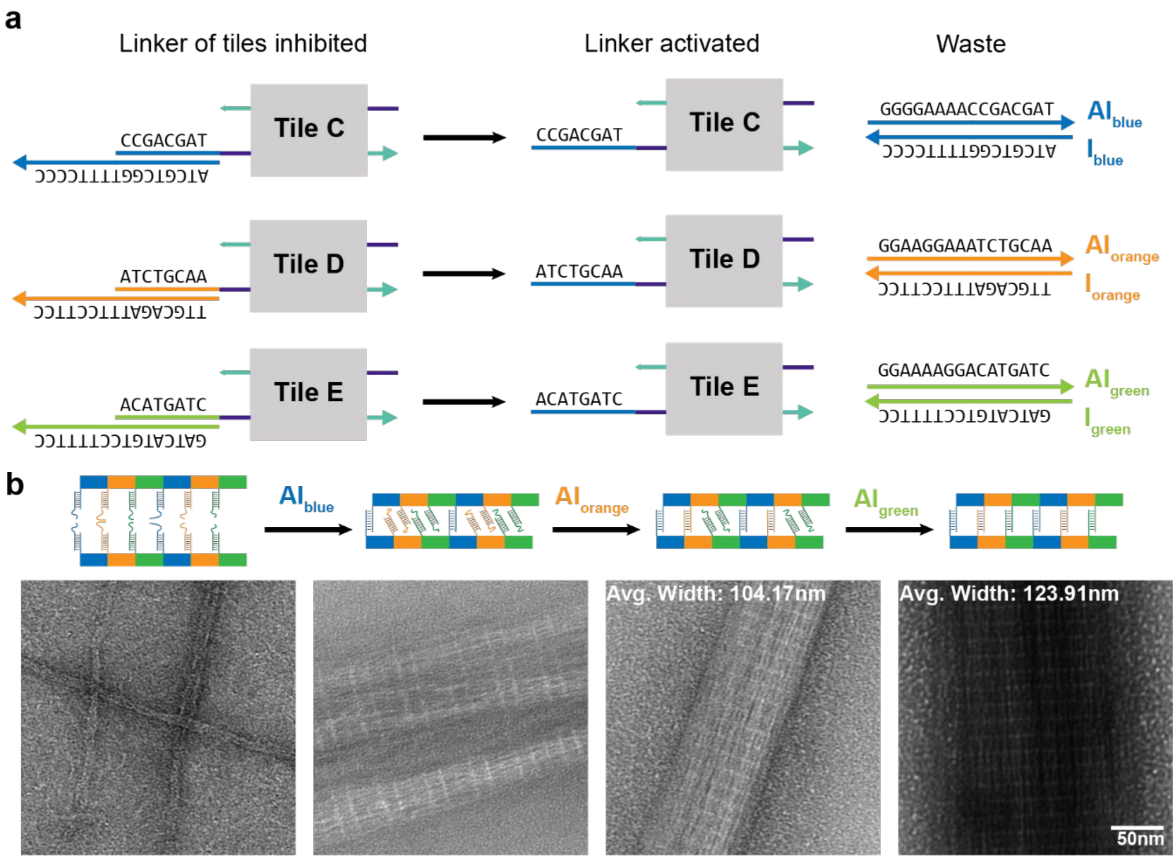

Figure S16. Sequentially activation of linkers for the transformation from DNA tubes to DNA bundles of prescribed features. (a) Schematics for linker inhibition and activation on various DNA tiles. (b) Representative TEM images showing the transformation result at each stage. In leftmost sample, no linker is activated. So, the assembled structure is normal nanotube. The second sample formed loosely bound bundle-like structures. The average width of the latter 2 groups were measured and labeled in the TEM images (N=25). All figures share the same scale bar of 50 nm.

Table S1. Sequences for DNA strands

Tile	Strand Name	Strand Sequence
A	S1	CTCAGTGGACAGCCGTTCTGGAGCGTTGGACGAAACT
	S2	GTTAGATAGAGCACCACTGAGAGGTA
	S3	CCAGAACGGCTGTGGCTAAACAGTAACCGAAGCACCAACGCT
	S4 to tile A	CTAACAGTTTCGTGGTCATCGTACCT
	S5	CGATGACCTGCTTCGGTTACTGTTTAGCCTGCTCTAT
	S2 polyT linker	TTTTTTT GTTAGATAGAGCACCACTGAGAGGTA
	S2 CGTTT linker	CGTTT GTTAGATAGAGCACCACTGAGAGGTA
	S2 CGCGTTT linker	CGCGTTT GTTAGATAGAGCACCACTGAGAGGTA
	S2CGCGCGTTT linker	CGCGCGTTT GTTAGATAGAGCACCACTGAGAGGTA
	S2 Varied T linker	CGCGTn GTTAGATAGAGCACCACTGAGAGGTA
	S2 ATCTGCA linker	ATCTGCA GTTAGATAGAGCACCACTGAGAGGTA
	S2 CGCAGCA linker	CGCAGCA GTTAGATAGAGCACCACTGAGAGGTA
	S2 TCGTCGT linker	TCGTCGT GTTAGATAGAGCACCACTGAGAGGTA
	S2 TTTCGCG linker	TTTCGCG GTTAGATAGAGCACCACTGAGAGGTA
	S2 GCTAACT linker	GCTAACT GTTAGATAGAGCACCACTGAGAGGTA
	S2 ATCTACA linker	ATCTACA GTTAGATAGAGCACCACTGAGAGGTA
	S2 TGGTATT linker	TGGTATT GTTAGATAGAGCACCACTGAGAGGTA
	S2 TTTTTCG linker	TTTTTCG GTTAGATAGAGCACCACTGAGAGGTA
	S2 CGTTTTT linker	CGTTTTT GTTAGATAGAGCACCACTGAGAGGTA
	S2 ACGTACGT linker	ACGTACGT GTTAGATAGAGCACCACTGAGAGGTA
	S2 CGCGCGT linker	CGCGCGT GTTAGATAGAGCACCACTGAGAGGTA
	S2 CCGACGAT linker	CCGACGAT GTTAGATAGAGCACCACTGAGAGGTA
	S2 inner linker	GTCTGAAATACTACGGGAATATCTAA TTTGCGC
	S3 CGCGTTT linker	CGCGTTT CCAGAACGGCTGTGGCTAAACAGTAACCGAAGCACCAACGCT
	S4 to tile B	CAGACAGTTTCGTGGTCATCGTTAGA
B	S1	TATTCCCGACCGCTTGCCTATGAATCCTTACCATGCC
	S2	GTCTGAAATACTACGGGAATATCTAA
	S3	ATAGGCAAGCGGTGCTCGTTGAGTCATCTGCTTCAAGGATTC
	S4 to tile B	CAGACGGCATGGTGTAGGGAGTTAGA
	S5	CTCCCTACGAAGCAGATGACTCAACGAGCTAGTATTT
	S2 polyA linker	AAAAAAA GTCTGAAATACTACGGGAATATCTAA
	S4 to tile C	CGTGCGGCATGGTGTAGGGAGTCATA
C	S1	CTCAGTGGACAGCCGTTCTGGAGCGTTGGACGAAACT
	S2	GTTAGATAGAGCACCACTGAGAGGTA
	S3	CCAGAACGGCTGTGGCTAAACAGTAACCGAAGCACCAACGCT
	S4 to tile C	CTAACAGTTTCGTGGTCATCGTACCT
	S5	CGATGACCTGCTTCGGTTACTGTTTAGCCTGCTCTAT
	S4 to tile D	CAGACAGTTTCGTGGTCATCGTTAGA
	S2 CCGACGAT linker	CCGACGAT GTTAGATAGAGCACCACTGAGAGGTA

D	S1	TATTCCCGACCGCTTGCCTATGAATCCTTACCATGCC
	S2	GTCTGAAATACTACGGGAATATCTAA
	S3	ATAGGCAAGCGGTGCTCGTTGAGTCATCTGCTTCAAGGATTC
	S4 to tile D	CAGACGGCATGGTGTAGGGAGTTAGA
	S5	CTCCCTACGAAGCAGATGACTCAACGAGCTAGTATTT
	S2 ATCTGCAA linker	ATCTGCAA GTCTGAAATACTACGGGAATATCTAA
	S4 to tile E	CGTGCGGCATGGTGTAGGGAGTCATA
E	S1	GAATAAATCTTAGTAACCAACTGACCGACATGCCATC
	S2	GCACGAGTGGAAGATTTATTCTATGA
	S3	GTTGGTTACTAAGGCGGATACCGCGTCCCTGCTTGTCGGTCA
	S4 to tile E	CTAACGATGGCATTGAGACTTTACCT
	S5	AAGTCTCAAAGCAGGGACGCGGTATCCGCCTTCCACT
	S2 ACATGATC linker	ACATGATC GCACGAGTGGAAGATTTATTCTATGA
	S4 to tile C	CTAACGATGGCATTGAGACTTTACCT
F	S1	CTCAGTGGACAGCCGTTCTGGAGCGTTGGACGAAACT
	S2	GTTAGATAGAGCACCACTGAGAGGTA
	S3	CCAGAACGGCTGTGGCTAAACAGTAACCGAAGCACCAACGCT
	S4 to tile F	CTAACAGTTTCGTGGTCATCGTACCT
	S5	CGATGACCTGCTTCGGTTACTGTTTAGCCTGCTCTAT
	S4 to tile G	CAGACAGTTTCGTGGTCATCGTTAGA
	S2 ATCTGCA linker	ATCTGCA GTTAGATAGAGCACCACTGAGAGGTA
G	S1	TATTCCCGACCGCTTGCCTATGAATCCTTACCATGCC
	S2	GTCTGAAATACTACGGGAATATCTAA
	S3	ATAGGCAAGCGGTGCTCGTTGAGTCATCTGCTTCAAGGATTC
	S4 to tile G	CAGACGGCATGGTGTAGGGAGTTAGA
	S5	CTCCCTACGAAGCAGATGACTCAACGAGCTAGTATTT
	S4 to tile F	CTAACGGCATGGTGTAGGGAGTACCT
	S2 GTCAACT linker	GTCAACT GTCTGAAATACTACGGGAATATCTAA
H	S1	GAATAAATCTTAGTAACCAACTGACCGACATGCCATC
	S2	GCACGAGTGGAAGATTTATTCTATGA
	S3	GTTGGTTACTAAGGCGGATACCGCGTCCCTGCTTGTCGGTCA
	S4 to tile H	CTAACGATGGCATTGAGACTTTACCT
	S5	AAGTCTCAAAGCAGGGACGCGGTATCCGCCTTCCACT
	S2 AATACCA linker	AATACCA GCACGAGTGGAAGATTTATTCTATGA
	S4 to tile I	GATGAGATGGCATTGAGACTTCACGG
I	S1	CAGATTATCGGTGTCCGCACTAAATACCTGTTACTGA
	S2	TCATCCTCTATGGATAATCTGCCGTG
	S3	AGTGCGGACACCGACAACGACACCACATTGGGCTAGGTATTT
	S4 to tile I	GATGATCAGTAACTTGAGCGTCACGG
	S5	ACGCTCAAAGCCCAATGTGGTGTCGTTGTCCATAGAG
	S2 AGTTGAC linker	AGTTGAC TCATCCTCTATGG ATAATCTGCCGTG
	S4 to tile H	CGTGCTCAGTAACTTGAGCGTTCATA

J	S1	CTCAGTGGACAGCCGTTCTGGAGCGTTGGACGAAACT
	S2	GTTAGATAGAGCACCACTGAGAGGTA
	S3	CCAGAACGGCTGTGGCTAAACAGTAACCGAAGCACCAACGCT
	S4 to tile J	CTAACAGTTTCGTGGTCATCGTACCT
	S5	CGATGACCTGCTTCGGTTACTGTTTAGCCTGCTCTAT
	S4 to tile K	CAGACAGTTTCGTGGTCATCGTTAGA
	S2 CGCGTTT linker	CGCGTTT GTTAGATAGAGCACCACTGAGAGGTA
K	S1	TATTCCCGACCGCTTGCCTATGAATCCTTACCATGCC
	S2	GTCTGAAATACTACGGGAATATCTAA
	S3	ATAGGCAAGCGGTGCTCGTTGAGTCATCTGCTTCAAGGATTC
	S4 to tile K	CAGACGGCATGGTGTAGGGAGTTAGA
	S5	CTCCCTACGAAGCAGATGACTCAACGAGCTAGTATTT
	S4 to tile L	GATGAGGCATGGTGTAGGGAGCACGG
	S2 GNP handle	GTCAACTCGTA GTTAGATAGAGCACCACTGAGAGGTA
	S2 STV handle	5'Biotin-TTTT GTTAGATAGAGCACCACTGAGAGGTA
L	S1	CAGATTATCGGTGTCCGCACTAAATACCTGTTACTGA
	S2	TCATCCTCTATGGATAATCTGCCGTG
	S3	AGTGCGGACACCGACAACGACACCACATTGGGCTAGGTATTT
	S4 to tile L	GATGATCAGTAACTTGAGCGTCACGG
	S5	ACGCTCAAAGCCCAATGTGGTGTCGTTGTCCATAGAG
	S4 to tile M	CAAAGTCAGTAACTTGAGCGTCGTCG
	S2 CGTTTCG linker	CGTTTCG TCATCCTCTATGGATAATCTGCCGTG
M	S1	GATGCGATATCAGGAAGTTACTCACAGCAGTGGAGCG
	S2	CTTTGGCCTCCAGATCGCATCCGACG
	S3	GTAACTTCCTGATTCATAATAGCCGTAGGTGTCTTGCTGTGA
	S4 to tile M	CAAAGCGCTCCACTAGAGCGTCGTCG
	S5	ACGCTCTAAGACACCTACGGCTATTATGACTGGAGGC
	S4 to tile J	CTAACCGCTCCACTAGAGCGTTACCT
	S2 GNP handle	ATCTACAGGCACTTTGGCCTCCAGATCGCATCCGACG
	S2 STV handle	5'Biotin-TTTTCTTTGGCCTCCAGATCGCATCCGACG

Reference

[1] B. Wei, Z. Wang, Y. Mi, Uniquimer: A de Novo DNA Sequence Generation Computer Software for DNA Self-assembly, In: Mao, C., Yokomori, T. (eds) DNA Computing. DNA **2006**. Lecture Notes in Computer Science, vol 4287. Springer, Berlin, Heidelberg.

[2] J. N. Zadeh, C. D. Steenberg, J. S. Bois, B. R. Wolfe, M. B. Pierce, A. R. Khan, R. M. Dirks, N. A. Pierce, *J Comput Chem* **2011**, 32, 170.

[3] Y. Hao, Y. J. Li, L. Song, Z. X. Deng, *J Am Chem Soc* **2021**, 143, 3065.

[4] P. W. Rothemund, A. Ekani-Nkodo, N. Papadakis, A. Kumar, D. K. Fygenson, E. Winfree, *J Am Chem Soc* **2004**, 126, 16344.

[5] L. N. Green, H. K. K. Subramanian, V. Mardanlou, J. Kim, R. F. Hariadi, E. Franco, *Nat Chem* **2019**, 11, 510.
